# Enhancing the Pressure-Sensitive Electrical Conductance of Self-Assembled Monolayers

**DOI:** 10.1021/acsami.4c15796

**Published:** 2024-11-19

**Authors:** Xintai Wang, Asma Alajmi, Zhangchenyu Wei, Mohammed Alzanbaqi, Naixu Wei, Colin Lambert, Ali Ismael

**Affiliations:** † College of Chemistry and Materials Engineering, Wenzhou University, Wenzhou, Zhejiang 325035 China; ‡ Physics Department, Lancaster University, Lancaster LA1 4YB, U.K.; § Zhejiang Mashang GM2D Research Institute, Cangnan, Wenzhou, Zhejiang 325800, China; ⊥ Department of Physics, College of Science and Humanities in Al-Kharj, Prince Sattam Bin Abdulaziz University, Al-Kharj 11942, Saudi Arabia; # Physics Department, College of Science and Arts in Rabigh, King Abdulaziz University, 344 Rabigh, Saudi Arabia

**Keywords:** Gauge factor, Tunnelling decay, Penetration, Self-Assembled Monolayers, Atomic Force Microscopy

## Abstract

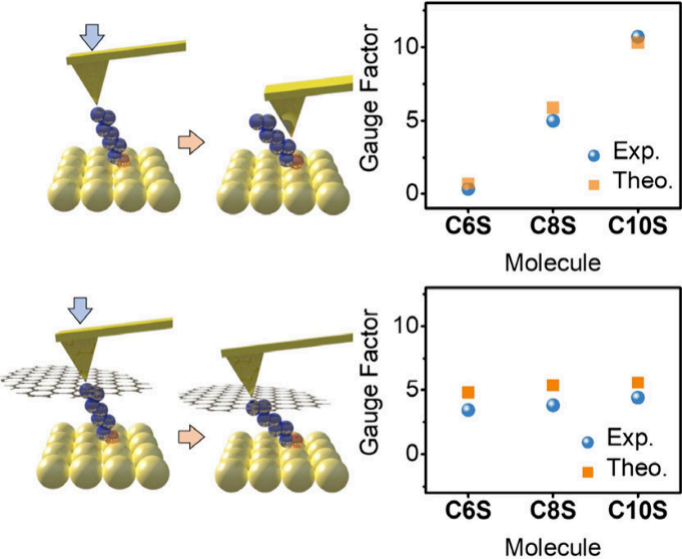

The inherent large HOMO–LUMO gap of alkyl thiol (CnS) self-assembled monolayers (SAMs) has limited their application in molecular electronics. This work demonstrates significant enhancement of mechano-electrical sensitivity in CnS SAMs by external compression, achieving a gauge factor (GF) of approximately 10 for C10S SAMs. This GF surpasses values reported for conjugated wires and DNA strands, highlighting the potential of CnS SAMs in mechanosensitive devices. Conductive atomic force microscopy (cAFM) investigations reveal a strong dependence of GF on the alkyl chain length in probe/CnS/Au junctions. This dependence arises from the combined influence of molecular tilting and probe penetration, facilitated by the low Young’s modulus of alkyl chains. Theoretical simulations corroborate these findings, demonstrating a shift in the electrode Fermi level toward the molecular resonance region with increasing chain length and compression. Introducing a rigid graphene interlayer prevents probe penetration, resulting in a GF that is largely independent of the alkyl chain length. This highlights the critical role of probe penetration in maximizing mechano-electrical sensitivity. These findings pave the way for incorporating CnS SAMs into mechanosensitive and mechanocontrollable molecular electronic devices, including touch-sensitive electronic skin and advanced sensor technologies. This work demonstrates the potential of tailoring mechanical and electrical properties of SAMs through molecular engineering and interface modifications for optimized performance in specific applications.

## Introduction

The ability to tune the electrical conductance of single molecules by modifying their conformation, either intrinsically^1−4^ or extrinsically,^5−8^ has been demonstrated in a number of studies. Recently, the ability to manipulate electrical conductance and thermoelectric properties of self-assembled monolayers (SAMs) by applying external pressure has been demonstrated.^5,9−11^ Other notable examples include mechanical control of redox-active chromophores,^12−14^ mechanical switching via control of the metal–molecule contact geometry or through stereo electronic effects,^15−18^ and mechanically induced spin-state switching.^19−21^ Conductive atomic force microscopy (cAFM), a nanoscale electric measurement technique employing probe as source and bottom substrate as drain, is effective for electromechanical investigations.^10,22^ This method enables extraction of nanomechanical properties of SAMs, while simultaneously mapping the electrical or thermoelectrical response, thus opening the way to designing novel pressure-sensitive organic materials, with potential applications to sensors,^23−25^ microelectromechanical systems (MEMs),^26^ human–computer interaction systems (HCIs),^27^ and power harvesters.^28^

In a high-performance pressure-sensitive material, an important parameter, which should be maximized, is the gauge factor (GF), defined as the relative change in electrical conductance divided by displacement normalized to the molecular length.^29−31^ Recently, junctions consisting of molecular wires such as DNA strands,^32^ polyaniline,^33^ and oligo-(phenylene-ethylene)s^34^ have been investigated, with reported GFs in the range of 0.1–5. In this work, we demonstrate that a significant improvement in GF can be achieved using cheaper, easier to synthesize, and readily available alkane-thiol molecules.

Recent intensive investigations have focused on the relationship between compression and electrical behavior in molecular wires, utilizing techniques such as mechanical break junction (MBJ) and conductive atomic force microscopy (CAFM).^11,28,34^ The former operates on single-molecule wires, while the latter is used for well-ordered molecular arrays such as self-assembled monolayers (SAMs). CAFM studies of different types of SAMs have revealed electric tunability under applied compression forces. These tunable properties are often attributed to molecular tilting under high pressure, which when probed by a CAFM tip moved toward the bottom electrode, results in a shift in the Fermi level and molecular orbital alignment, thereby regulating electron transport behavior.^9,11,28,35,36^

**Figure 1:**
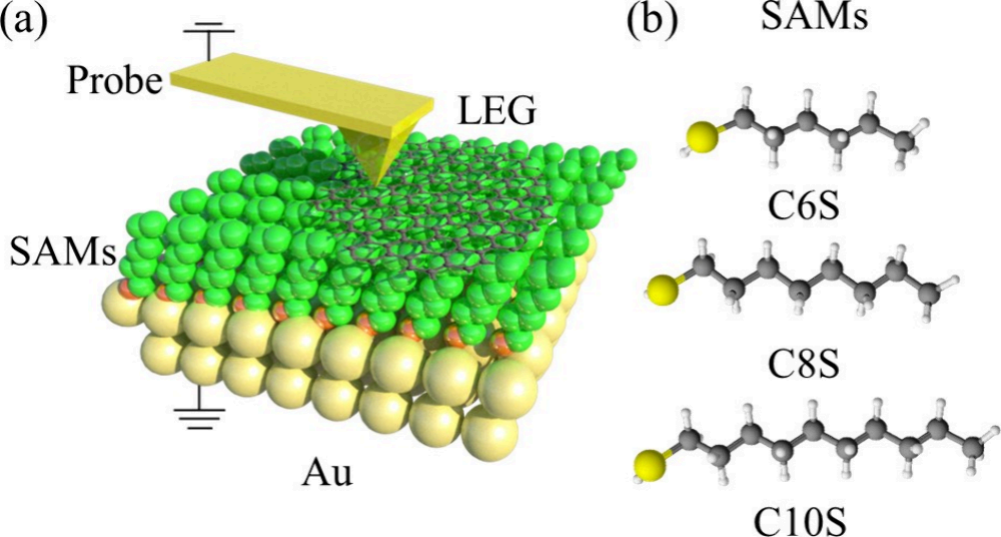
(a) Experiment setup and (b) SAMs molecule investigated in this work.

SAMs formed from alkyl thiol molecules (CnS), comprising *n* carbons and a single thiol, have been extensively studied due to their highly ordered array formation on gold substrates. These SAMs have not been considered ideal for use in mechanoelectrical devices, because of their large HOMO–LUMO gap, leading to an electron transmission probability, which is relatively insensitive to frontier-orbital shifts induced by molecular tilting.^1,37−39^ In this study, conductive atomic force microscopy (CAFM) investigations were carried out on CnS (*n* = 6, 8, and 10, Figure 1) systems assembled on a gold substrate, forming a probe/CnS/Au junction. A significant positive correlation between the number n of alkyl units and the mechanoelectrical sensitivity of the SAMs was observed. However, when a liquid phase exfoliated graphene (LEG) nano flake (NF) was introduced between the probe and the SAMs (forming a probe/LEG/CnS/Au junction), this trend was not observed. This result and our accompanying theoretical calculations strongly suggest that, in addition to molecular tilting, the penetration of the probe into the SAMs can enhance the mechanoelectrical behavior of CnS SAMs.

## Result and Discussion

**Figure 2:**
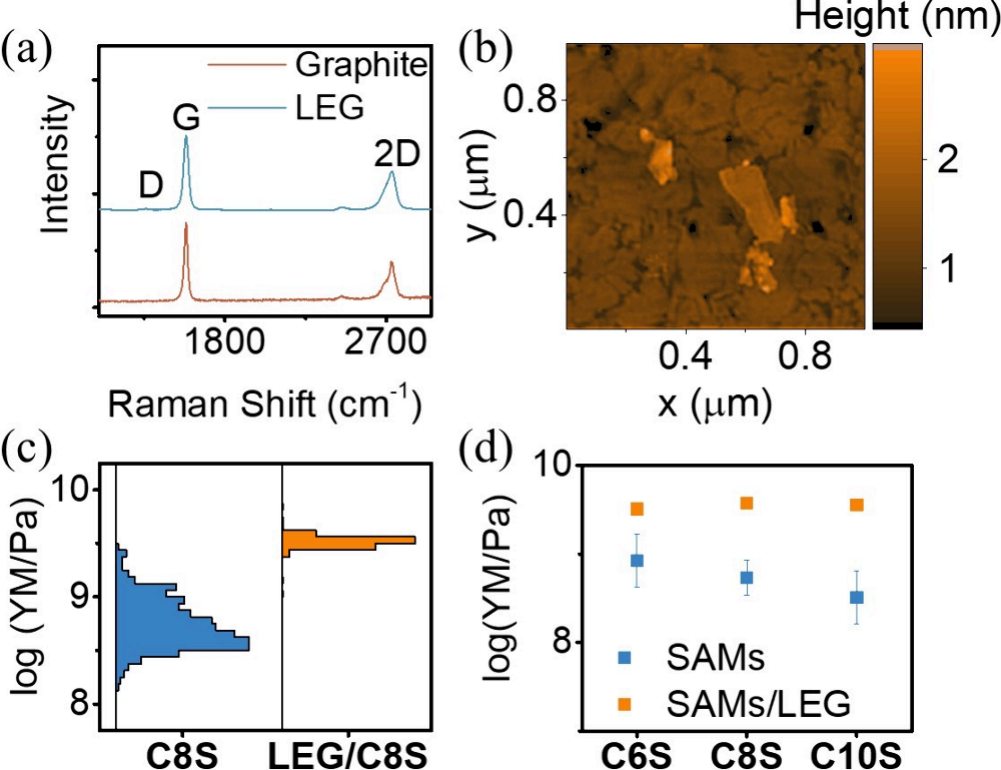
(a) Raman spectra of LEG and (b) an example of an AFM image while the flake was coated on SAMs. (c) Young’s modulus (YM) distribution of C8S SAMs with and without LEG coating and (d) the YM for different types of SAMs measured in this work.

High-quality graphene nanoflakes (LEG-NF) were prepared via liquid-phase exfoliation of graphite in Cyrene, a solvent with Hansen solubility parameters closely matching those of graphene. The thickness of the LEG-NF was determined as a function of exfoliation time using tapping-mode atomic force microscopy (AFM) (Supporting Information (SI), Figure S1). After 14 h of ultrasonication, the exfoliated flakes exhibited a thickness of 0.8–1.2 nm, indicating the successful isolation of predominantly monolayer graphene (SI, Figure S1d,e). Raman spectroscopy further confirmed the high quality of the exfoliated graphene. The Raman spectrum of LEG-NF displays the characteristic G and 2D peaks, as shown in Figure 2a. The absence of a D peak indicates that minimal defects or oxidation are introduced during the exfoliation process. Additionally, the suppression of the shoulder peak in the 2D region, commonly observed in bulk graphite due to interlayer interactions, suggests the presence of few-layer graphene.^40,41^

To investigate the influence of LEG-NF on the mechanical properties of self-assembled monolayers (SAMs), LEG-NF were deposited onto template-stripped gold (Au^TS^) substrates prefunctionalized with CnS SAMs (*n* = 6, 8, 10). The SAMs were prepared using a standard procedure (see the Experimental Section). Both experimental and theoretical evidence confirmed the robust immobilization of LEG-NF on the SAMs, attributed to the strong interaction between the alkyl chains of the SAMs and the graphene surface (Figure 2b, SI, Figure S10). Peak-force quantitative nanomechanical (PF-QNM) AFM was employed to determine the Young’s modulus (YM) of the SAMs, both with and without the LEG-NF top layer. The YM, a measure of material stiffness, was determined for CnS/Au SAMs (*n* = 6, 8, 10) to be 810, 570, and 330 MPa, respectively. These values are consistent with previously reported YM values for alkyl-thiol SAMs, which range from 100 MPa to 1 GPa.^36,39^Figure 2c displays the YM distribution of C8S/Au, where a relatively broad YM distribution was observed. Similar broad distributions were also observed in the C6S/Au and C10S/Au SAMs, as shown in SI, Figure S3b. The relatively broad YM distribution for C8S is attributed to the distribution of defects within the SAMs, which disrupt the hydrophobic interactions between the tails, leading to YM nonuniformity. This nonuniformity was further confirmed by quartz crystal microbalance (QCM) measurements described in the Experimental Section. Supporting Information, Figure S3a shows the molecular occupation area estimated from the QCM measurements. The QCM results reveal a comparable, yet slightly higher molecular occupation area (∼0.26, 0.24, and 0.21 nm^2^ for C6S, C8S, and C10S) compared to the theoretical value. This suggests the presence of some defects in the SAMs, resulting in a reduced number of molecules per unit area and a larger molecular occupation area. A decreasing trend in molecular occupation area is observed with increasing alkyl chain length, which is expected because longer alkyl tails enhance the hydrophobic interaction forces between the tails, facilitating the alignment of molecules in a closely packed manner. This, in turn, reduces the likelihood of defects occurring. Typically, a decrease in defects would be expected to result in higher YM values, as the molecules become more crystalline. However, the results of this work show an opposite trend: longer-tailed molecules have fewer defects but also exhibit lower YM values, as shown in Figure 2d. We hypothesize that this may be due to the Au substrate beneath the SAMs also affecting the measured YM values. Shorter-tailed molecules result in thinner films, which are more influenced by the underlying Au electrode.

The YM of the CnS/LEG/Au junctions was measured by positioning the AFM probe on top of monolayer graphene flakes (∼1 nm thickness). In contrast to the bare SAMs, the YM for the CnS/LEG/Au junctions remained relatively constant at 3.2, 3.7, and 3.5 GPa for *n* = 6, 8, and 10, respectively. This significant increase in stiffness compared with the bare SAMs highlights the dominant role of the graphene structure in determining the overall mechanical properties. The sp^2^ hybridized carbon atoms in graphene form a robust network of strong σ bonds within the plane and delocalized π bonds above and below the plane, contributing to its exceptional mechanical strength and stiffness. The insensitivity of the YM to the underlying alkyl chain length shown in Figure 2d further supports this conclusion, suggesting that the mechanical properties of the CnS/LEG/Au junctions are primarily governed by the graphene top layer. As a reference, the YM for LEG/Au was measured to be 6.5 GPa. This value is comparable yet slightly higher than that of LEG/SAMs/Au. The reason for this increase lies in the fact that, within the LEG/SAMs/Au system, the YM of LEG is significantly higher than that of the SAMs, thereby predominantly influencing the measured YM. Conversely, in the LEG/Au system, the higher YM of Au compared to LEG, coupled with the very thin nature of the LEG layer (∼1 nm), allows the underlying Au substrate to contribute for increasing the measured YM. Moreover, compared with CnS/Au, much narrower YM distribution of LEG/CnS was observed as shown in Figure 2d, this was due to the uniformed honey-combo structure of different graphene flakes.

The electrical transport behavior of SAMs was investigated using conductive atomic force microscopy (CAFM), as detailed in the Experimental Section. A noble-metal-coated AFM probe served as the source, while the gold substrate acted as the drain, with the SAM (or SAMs/LEG) serving as the functional component. Junctions were formed by placing the probe on and out of the graphene-coated areas, resulting in probe/CnS/Au and probe/LEG/CnS/Au junctions. The electron transport properties of these junctions were measured by applying a bias voltage between the source and drain and recording the resulting current. The contact area between the probe and sample can be calculated and scaled using the Hertz model:^28,42−44^
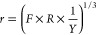
1
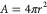
2where *A* is the probe-sample contact area, *r* is the contact radius, *F* is the loading force between the probe and sample, *R* is the radius of the probe estimated via scanning electron microscope, and *Y* is the junction effective modulus defined as
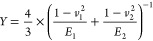
3*E*_1_ is the YM of the SAMs or graphene coated SAMs, measured via QNM-AFM as described in previous section; *E*_2_ is the YM of AFM probe, which is ∼10^11^ N/m^2^ according to previous publication, and *v*_1_ (∼0.33) and *v*_2_ (0.3) denote the Poisson’s ratios of the respective materials.^44^

The electrical conductance of SAMs in their natural form, without deformation induced by a probe loading force, was used as a reference for investigating mechanoelectrical behavior. The nanoscratching technique, as detailed in the experimental section, was employed to ascertain the film thickness, *d*, of the SAMs. The measurements yielded values of 0.71 ± 0.2, 0.9 ± 0.2, and 1.15 ± 0.1 nm for CnS/SAMs, where *n* is 6, 8, and 10, respectively. The molecular length, *L*, of CnS was approximated through density functional theory (DFT) calculations. The tilt angle of SAMs molecule respect to the perpendicular plane toward Au substrate, θ, without external pressing force was derived using the following equation:^28,42,43^
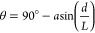
4The calculated tilt angles for CnS/SAMs with *n* = 6, 8, and 10 were 34°, 37°, and 31°, respectively (SI, Figure S4). These angles are consistent with previously reported data, suggesting that thiol-based molecules typically exhibit a tilt angle ranging from 30° to 60°.^45,46^ Although a positive correlation between the tilt angle and molecular occupation area was anticipated given that molecules in defect regions would be expected to tilt more than those in well-ordered areas, this relationship was not observed in our study. This discrepancy is attributed to the uncertainty in the film thickness measurements, which overshadows the influence of defects.

**Figure 3:**
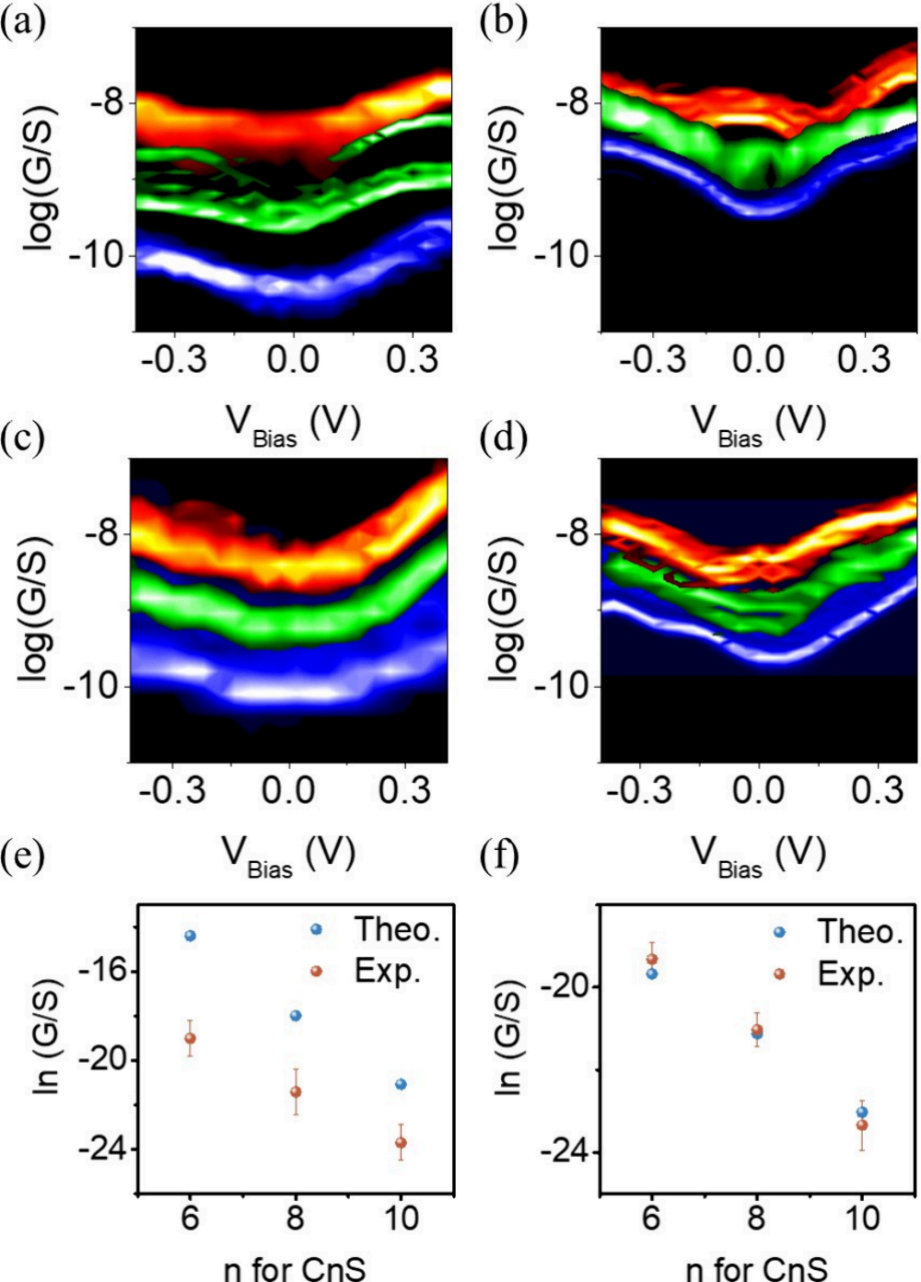
(a) Statistical GV (scaled to SMA) curve for probe/CnS/Au (2 nN), (b) probe/CnS/Au (10 nN), (c) probe/LEG/CnS/Au (2 nN), and (d) probe/LEG/CnS/Au (10 nN). Red, green, and blue curve represents C6S, C8S, and C10S, respectively. (e) Agreement between theory and experiment for probe/CnS/Au junction (2 nN). (f) Probe/LEG/CnS/Au junction (2 nN).

For the cAFM measurement, the molecular configuration was determined by the loading force from the probe. A small loading force preserves the SAM’s natural configuration but results in poor interfacial contact and measurement reliability, whereas a high loading force yields the opposite effect. In this study, a force of 2 nN was employed as the reference measurement to achieve a compromise between the preservation of SAMs configuration and the reliability of the measurements. At this force, the differential conductance at sweeping bias (GV curve) for the probe/CnS/Au junction was obtained, as explained in the Experimental Section, and with statistical results shown in Figure 3a. Each GV curve was statistically obtained from over 80 measurements from at least 10 different locations. The results were scaled to conductance per single-molecule occupation area, which was used to compare to the results from our theoretical calculations. The number of contact molecules under the probe was estimated from *A*/SMA, where *A* is the contact area calculated via eq 2, and SMA is the single molecular occupation area for alkyl thiol, which obtained from QCM measurement explained in experiment section.

The scaled differential conductance, *G*, per single molecular occupation area for probe/CnS/Au was 7.9 × 10^–9^, 6.5 × 10^–10^, and 5.9 × 10^–11^ S, for *n* is 6, 8, and 10, respectively (Figure 3a). This value is about 30–40 times lower than the single molecular conductance for the same system from DFT calculations (Figure 3e). A lower measured conductance compared with theory was expected because of the complicated metal–organic interface between the probe and SAMs molecules. When viewing the molecules beneath the probe as a number of parallel resistors, only a small portion of those resistors forms electrical contact and contributes to the measured conductance. The same phenomenon has also been reported in other works.^47^

Figure 3e shows the scaled differential conductance vs alkyl thiol chain length. The relationship follows the trend:
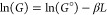
5where *G* is the measured conductance, *L* is the molecular length obtained from DFT calculation, which is 0.87, 1.11, and 1.36 nm for C6S, C8S, and C10S respectively, *G*° is the contact conductance at *L* = 0, and β is the tunneling decay factor related to the tunneling barrier height (Figure 3e). The linear relationship between ln(*G*) and *L* is because the high energy barrier presented by self-assembled monolayers (SAMs) between the probe and gold surface necessitates the electron tunneling regime, thus, the electron wave function decays exponentially within the barrier region.

The measured β value for Probe/CnS/Au was 8.3 nm^–1^. A large β value is expected in this system due to the large HOMO–LUMO gap of the alkyl chain. Compared with theoretical simulations, which predict β to be 10 nm^–1^, this value is distinguishably lower. We attribute the observed discrepancy to the inaccuracy of the probe/CnS/Au measurements. The intricate metal/CnS interface likely impacts the number of molecules effectively forming a junction with the probe, thereby introducing uncertainty into the measured results. The substantial variation in YM values obtained from probe/CnS/Au measurements further supports this hypothesis. This uncertainty, attributed to the complex probe/SAMs interface, has also been reported in previous studies utilizing cAFM or EGaIn techniques.^48,49^ It is important to note that this work utilized a Pt-coated probe as the top electrode, resulting in a junction with dissimilar top (Pt) and bottom (Au) electrode materials. While asymmetric electrode materials have been reported to influence molecular transport properties,^50^ a significant impact is not anticipated in this study. The work functions of Pt (∼5.6 eV)^51^ and Au (∼5.3 eV)^52^ are similar, making a substantial shift in electrode Fermi level unlikely. Moreover, the large HOMO–LUMO gap of the CnS/Au system positions the electrode Fermi level energetically far from the frontier orbitals, further minimizing the impact of any Fermi level shift arising from the asymmetric electrodes.

For the probe/LEG/CnS/Au junction, the β was expected to be smaller because of the difference in the energetic positions of their frontier orbitals relative to the electrode Fermi energy (*E*_F_), as reported in previous publications.^53^ In this work, our theoretical calculations also indicated a decrease in the β factor (from 10 to 8.5 nm^–1^) after adding a graphene interlayer.

The probe/LEG/CnS/Au junction was experimentally formed by positioning an AFM probe on top of the monolayer LEG flakes. Electrical measurements, identical to those conducted on the probe/CnS/Au junction, were performed, with results displayed in Figure 3c. Notably, the graphene flake size in this work (∼200 nm on average) is significantly higher than the probe’s radius of curvature (∼10 nm). Due to graphene’s high conductivity, we anticipated two potential electrical transport pathways for probe/LEG/SAM/Au junction. The first pathway involves the probe/LEG (cross-plane)/SAMs (beneath the probe)/Au, whereas the second pathway involves the probe/LEG (in-plane)/SAMs (beneath the graphene but not beneath the probe)/Au. The first pathway is independent of LEG flake size, whereas the second pathway is LEG flake size dependent. Supporting Information, Figure S5, collected the measured conductance of probe/LEG/SAM/Au junction with different LEG flake size at 2 nN. Near-independence of measured conductance on the graphene flake size suggests that even at relative low loading force, the first pathway is still dominating the electron transport process, indicating that SAMs beneath the graphene but without external force from the probe do not establish effective electrical contact. The measured differential conductance, after scaling to a single molecular area for probe/CnS/Au, was 5.7 × 10^–9^ S, 9.8 × 10^–10^ S, and 8.10^–11^ S for *n* = 6, 8, and 10, respectively. Interestingly, unlike probe/CnS/Au junctions, where experimental and theoretical results differed significantly (by 30–40 times), Probe/LEG/CnS/Au junctions exhibited good agreement between experimental and theoretical molecular conductances (Figure 3f and Table 1). This enhanced agreement is attributed to the presence of a graphene interlayer, which transforms the molecule/metal interface into a composite of molecule/LEG and LEG/metal interfaces. The molecule/graphene interlayer exhibits stability due to the relatively high coupling energy between the alkyl unit and the graphene flake, as illustrated in SI, Figure S10. The probe/LEG interface is also deemed reliable, owing to the uniform structure of the graphene layer, which is further supported by the narrow distribution of the measured YM and the absence of a D peak in the Raman spectra. Consequently, this interfacial modification effectively addresses the issue of contact uncertainty and enhances the precision of the measurements. Consequently, the β factor for the measured system was 8.1 nm^–1^, closely aligning with the theoretically predicted value (Table 1).

**Table 1:** Experiment and Theory (Models 1 and 2) of Determining the Molecular Conductance for the Studied Junctions with and without LEG Interlayer

	exp^a^	theo mod 1	fit^b^	theo mod 2	fit
no LEG					
C6S-2 nN	1.1 × 10^–4^	2.3 × 10^–3^	3	2.0 × 10^–3^	3
C8S-2 nN	8.4 × 10^–6^	2.0 × 10^–4^	3.2	7.3 × 10^–4^	4.4
C10S-2 nN	8.1 × 10^–7^	4.6 × 10^–5^	4	1.6 × 10^–4^	5.3
C6S-10 nN	1.3 × 10^–4^	3.7 × 10^–3^	3.3	3.0 × 10^–3^	3.1
C8S-10 nN	3.6 × 10^–5^	8.6 × 10^–4^	3.2	1.8 × 10^–3^	3.9
C10S-10 nN	6.3 × 10^–6^	2.1 × 10^–4^	3.5	4.9 × 10^–4^	4.4
LEG					
C6S-2 nN	7.5 × 10^–5^	3.7 × 10^–5^	0.6	NA	
C8S-2 nN	1.1 × 10^–5^	8.7 × 10^–6^	0.2		
C10S-2 nN	1.1 × 10^–6^	1.3 × 10^–6^	0.2		
C6S-10 nN	1.1 × 10^–4^	1.1 × 10^–4^	0		
C8S-10 nN	1.7 × 10^–5^	2.3 × 10^–5^	0.3		
C10S-10 nN	3.2 × 10^–6^	4.1 × 10^–6^	0.2		

a Both experiment and theory conductance are in *G*/*G*_0_, where *G*_0_ is the quantum conductance defined as 2*e*^2^/*h*, *e* is the elementary charge = 1.6 × 10^–19^C, *h* is the Planck constant = 6.6 × 10^–34^ Js.

b Fit in the table is the parameter reflect the agreement between experiment result and theoretical predicted result, obtained via equation fit = |ln (*G*_Theo_/*G*_Exp_)|. Smaller fit value means better fitness between experimental measured and theoretical calculated result.

To investigate the mechanoelectrical tunability of CnS SAMs, the loading force between the sample and probe was controlled. This force dictates the distance (δ) between the probe and the bottom electrode, as defined by the equation via Johnson, Kendall, and Roberts (JKR) model:^54^
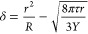
6*R* is the tip radius, *r* is the contact radius which is controllable by regulating the loading force as expressed in eq 1, *Y* is the effective modulus introduced in the previous section, and τ is the intrinsic surface energy parameter defiend as
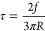
7where *f* is the tip/sample adhesion force obtained from the AFM force curve.

**Figure 4:**
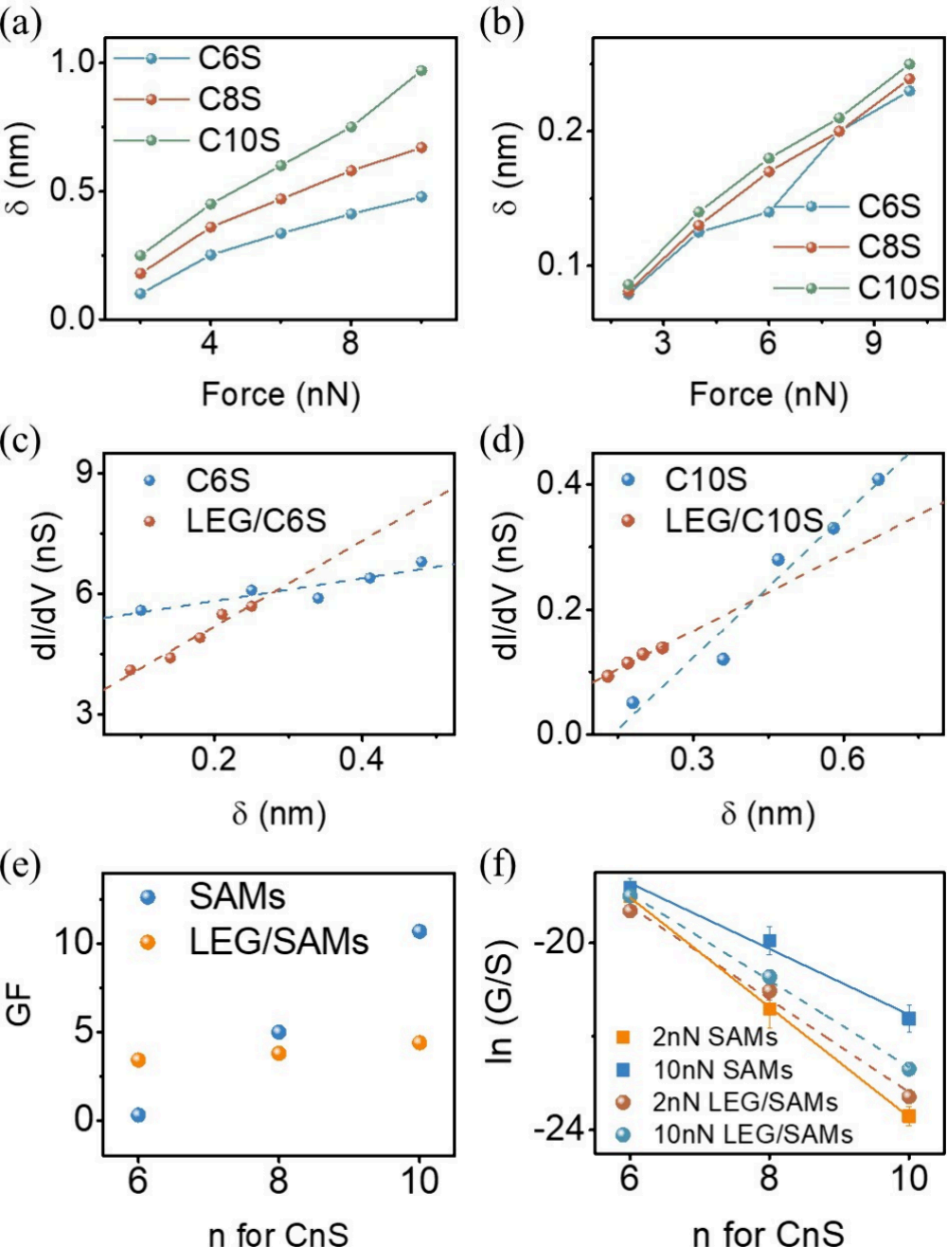
(a) the relationship between loading force of AFM and electrode displacement for probe/CnS/Au, (b) probe/LEG/CnS/Au, (c) measured SAMs conductance (scaled to SMA) vs electrode displacement for C10S, (b) measured SAMs conductance (scaled to SMA) for different SAMs, and (e) gauge factor (GF) for different SAMs. (f) Plot of experimentally obtained ln *G* vs *n* for CnS, at 2 nN and 10 nN with and without LEG protection.

Parts a and b of Figure 4 illustrate the electrode displacement (δ) as a function of the loading force for both probe/CnS/Au and probe/LEG/CnS/Au, calculated using eq 6. The top and bottom electrode displacement shifts in response to loading force are significantly influenced by the YM of the SAMs. Specifically, softer materials permit greater probe penetration at a given force, resulting in a more pronounced δ value for probe/CnS/Au compared to probe/LEG/CnS/Au.

The measured conductance at near-zero bias was recorded for all systems under an increasing loading force (Figure 3b,d). Using eq 2 to estimate the contact area between the probe and SAMs at different forces and incorporating the single-molecule occupation area obtained from QCM measurements, the measured conductance data scaled to a single molecular area (SMA). This scaling mitigates the influence of increasing contact area with rising force. In all cases, the scaled conductance increased with loading force. By correlating the loading force to electrode displacement (δ), a linear relationship between δ and scaled conductance emerged for all systems. Notably, for the short-chain molecule (C6S), the slope of scaled conductance versus electrode displacement was steeper for the probe/LEG/CnS/Au junction compared to the probe/CnS/Au junction (*n* = 6, Figure 4c). Conversely, the opposite trend was observed for the long-chain molecule, where probe/CnS/Au was steeper (*n* = 10, Figure 4d).

This slope reflects the gauge factor (GF) of the junction, a parameter used to characterize the piezoresistive response of a molecular wire. GF can be determined using the equation:^55^
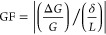
8where Δ*G* represents the change in measured conductance associated with the distance change (δ) between the probe and bottom electrode, *G* denotes the measured conductance at 2 nN loading force, and *L* represents the natural thickness of the SAM (see SI, Table S14). Notably, the GF can derive either from fraction of conductance change (Δ*G*/*G*)^55^ or resistance change (Δ*R*/*R*).^56^ In this work, (Δ*G*/*G*) was used for GF calculation, and for comparison, the GF result from Δ*R*/*R* is listed in SI, Table S13.

In this work, relative conductance change was used instead of relative resistance change for GF calculation, and the GF value obtained from relative resistance change is listed in SI, Table S13.

The calculated GFs values for probe/CnS/Au and probe/LEG/CnS/Au systems are presented in Figure 4e. For the probe/CnS/Au junction, the GF exhibited a strong dependence on the alkyl chain length, with values of 0.3, 5, and 10.7 for *n* = 6, 8, and 10, respectively. This indicates that electron transmission probability is more sensitive to electrode displacement shifts in longer-tailed molecules. In contrast, the GF for the probe/LEG/CnS/Au junction increased with alkyl chain length but was much less sensitive overall (GF = 3.4, 3.8, and 4.4 for *n* = 6, 8, and 10, respectively). The distinct GF values obtained from probe/CnS/Au and probe/LEG/CnS/Au, despite using the same CnS layer, can be attributed to the additional LEG layer, which significantly altered the transmission probability within the electrode Fermi region. This alteration includes a reduction in the broadening factor of the electron transmission node and a shift in the alignment of the electrode Fermi level relative to the molecular frontier orbitals, as illustrated in SI, Figure S17 and S18, thus changing the conductance/electrode displacement correlation.

**Figure 5:**
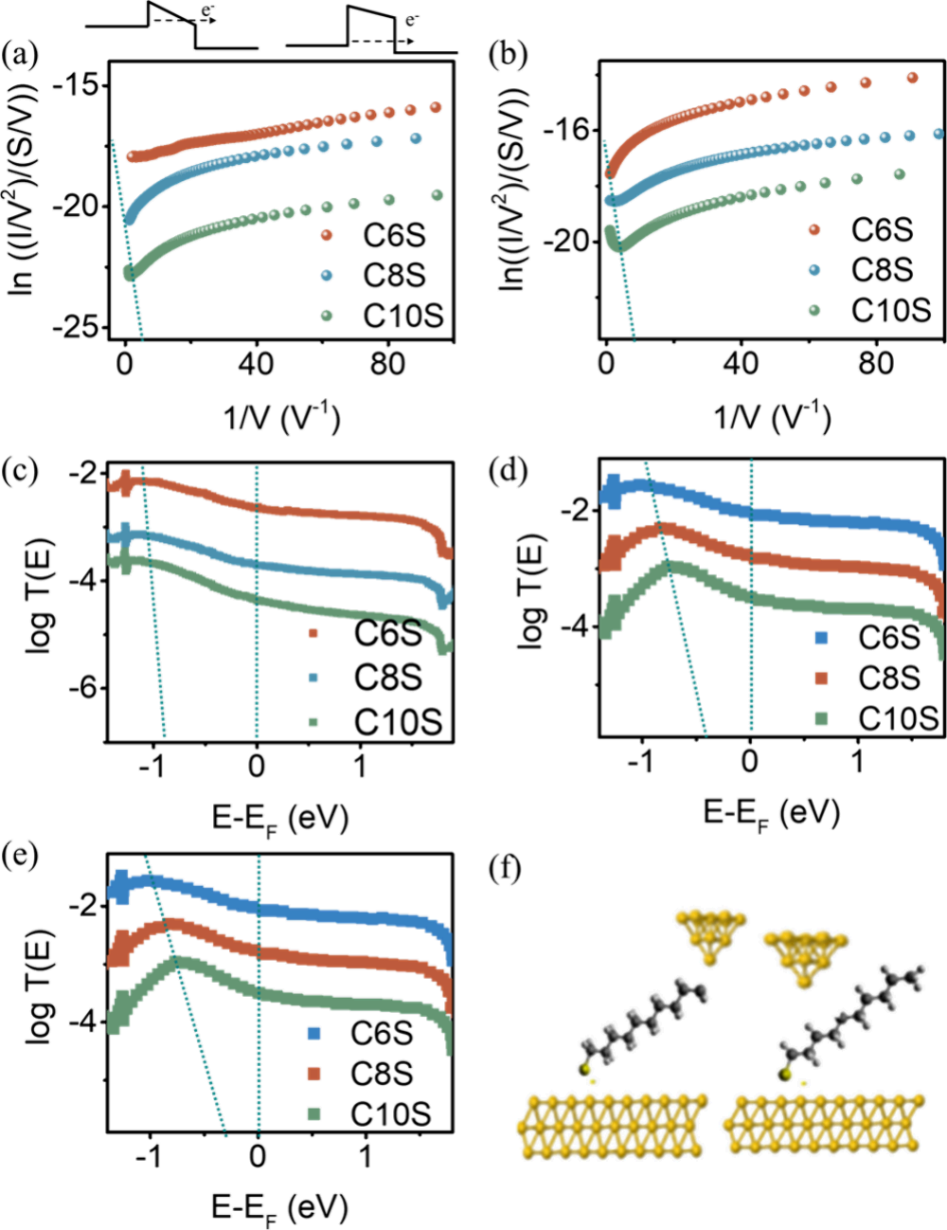
(a) Fowler–Nordheim plot for probe/CnS/Au junction at 2 nN (top panel an illustration of regime shifts from DT (right) to FNT (left)), (b) 10 nN, (c) theoretically calculated transmission curve for probe/CnS/Au at 2 nN, (d) 10 nN with Model 1, (e) 10 nN with Model 2, and (f) illustration for models 1 (left) and 2 (right).

Previous research revealed that while microscopic probe engaged toward bottom electrode for a SAMs system, the probe will slide and contact among different binding site of molecules.^57^ To elucidate this, two model systems were employed to describe the conductance shift with electrode displacement (Figure 5f). Model 1 assumes that the probe pressure tilts the SAMs at an angle of θ, maintaining contact with the terminal CH_3_ unit of the SAMs during compression (Figure 5f, left). Conversely, Model 2 assumes that probe compression leads to sliding into the SAMs, forming a junction by contacting the middle CH_2_ unit of the aliphatic chain (Figure 5f, right).

Previous studies have demonstrated that changes in electrode displacement can significantly alter the molecular dipole moment in a molecular junction, subsequently influencing the energetic alignment between molecular energy levels and the Fermi level.^58^ This energetic alignment can be estimated using a Fowler–Nordheim (FN) plot.^59,60^ This energetic alignment can be estimated by using a Fowler–Nordheim (FN) plot. While applying a low bias voltage, electron transport through self-assembled monolayers (SAMs) occurs via direct tunneling (DT) due to the presence of a high potential barrier. As the bias voltage increases, the substantial external electric field distorts the potential barrier into a triangular shape, thereby reducing the tunnelling barrier width, as depicted in the inset of Figure 5a. This reduction in barrier width alters the current–voltage relationship from I ∝ *V*, characteristic of DT, to *I* ∝ *V*^2^*e*^–*B*/*V*^, which is indicative of Fowler–Nordheim tunnelling (FNT). In the FNT spectra, a plot of ln(*I*/*V*^2^) versus 1/*V* is constructed; a linear relationship indicates FNT, while a logarithmic relationship suggests DT, in accordance with the aforementioned *I*–*V* relationship. The voltage at which the transition from one relationship to the other occurs is denoted as the transition voltage (*V*_*T*_). This *V*_*T*_ correlates with the energy offset between the electrode Fermi level and the nearest molecular orbital.^61,62^Figure 5a displays the FN plot for probe/CnS/Au at 2 nN. For *n* = 6 and 8, no clear transition point is observed within the measured voltage range (up to 1 V), indicating that electron transport is dominated by direct tunnelling. This suggests that the electrode Fermi level is energetically distant from the molecular frontier orbitals. In contrast, for *n* = 10, a transition point (the minimum point on the FN curve) is observed at *V*_*T*_ = 0.83 V. Employing the approximate relation from Baldea (*eV*_*T*_ ≈ 1.1φ),^61,62^ where φ represents the energy difference between the electrode Fermi level and the frontier orbital, yields a φ value of approximately 0.75 eV for probe/C10S/Au at 2 nN. Figure 5c depicts the theoretically calculated transmission curve for the probe/CnS/Au junction. This curve reveals that increasing alkyl chain length pushes the electron transmission node from the molecular frontier orbital closer to the Fermi level, explaining why *V*_*T*_ is observed only for longer-tailed molecules.

Increasing the loading force to 10 nN allows the probe to penetrate further toward the bottom electrode (eq 6), resulting in an increased scaled conductance. For C6S, the conductance increased by a factor of 1.2 when the force was increased from 2 nN to 10 nN. This increase was more pronounced as the molecular tail length increased (∼4 times for C8S and ∼8 times for C10S). The FN plots for probe/CnS/Au at 10 nN are shown in Figure 5b. The estimated φ values are >1 eV for C6S, 0.62 eV for C8S, and 0.41 eV for C10S, meaning longer tailed molecule the electrode Fermi is closer to the molecular frontier orbital.

For comparing the experimental results with the theory, the molecular tilt angles (θ) at different pressing forces were estimated using the equation:
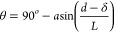
9where *d* is the film thickness measured via nanoscratching, *L* is the molecular length estimated from density functional theory (DFT) calculations, and δ is the probe penetration depth calculated using eq 6, which is dependent on both loading force and the YM of the monolayer.

Using this equation, the tilt angle for probe/CnS/Au at 10 nN was estimated to be approximately 74°. Parts d and e of Figure 5 show the theoretically calculated transmission curves for the probe/CnS/Au junction at 10 nN, considering both Model 1 and Model 2. Compared to the 2 nN case, the increased pressure shifts the electron transmission node from the molecular frontier orbital closer to the electrode Fermi level for both models, consistent with the FN plot results. As the electrode Fermi level approaches the closest molecular frontier orbital, the density of states becomes increasingly sensitive to the Fermi level alignment. For short-chain alkyl thiols like C6S, where the electrode Fermi level is situated far from the molecular frontier orbital and within the energy gap, the conductance shift is relatively insensitive to Fermi level shifts. Consequently, a low GF is observed under pressing force. Conversely, for longer molecules, because the electrode Fermi level is close to the frontier orbital, the increasing force pushed this Fermi from flat midgap region to steep resonance region, leading to a significant enhancement in electron transmission probability and a high GF. This shift in Fermi level alignment also affects the β factor for probe/CnS/Au at different forces. At 2 nN, the measured β factor was 8.3 nm^–1^, whereas at 10 nN, it decreased to 5 nm^–1^. Theoretical simulations corroborate this decreasing trend with increasing force, attributed to the electrode Fermi level shifting from the midgap region to the near-resonance region with increasing of chain length.

For reference, the probe/LEG/CnS/Au junction was also analyzed. In this system, the graphene layer acts as a protective barrier between the probe and the CnS molecule, preventing the penetration of the probe into the SAMs. Therefore, increasing the loading force affects only the molecular tilt angle (Model 1). As the loading force increased from 2 nN to 10 nN, the tilt angle for probe/LEG/CnS/Au shifted as well through estimation from eq 9 (35.1° to 44.3°, 34.1° to 46.3°, and 34.4° to 50.5° while *n* is 6, 8 and 10, respectively). At 10 nN, the measured scaled junction conductivity for probe/LEG/CnS/Au was 8 × 10^–9^ S, 1.3 × 10^–9^ S, and 2.4 × 10^–10^ S for *n* = 6, 8, and 10, respectively. These values are approximately 1.5 times higher than those observed at 2 nN. The β factor remained relatively constant between the 2 and 10 nN cases (8.1 for 2 nN and 7.8 for 10 nN). These findings are consistent with theoretical predictions (8.5 for 2 nN and 8.2 for 10 nN).

## Conclusion

The electron transport properties of alkyl thiol (CnS) self-assembled monolayers (SAMs) have been extensively studied, revealing limitations in their application within molecular electronics due to their inherent large HOMO–LUMO gap. This work demonstrates a significant advancement by achieving a high gauge factor (GF) of approximately 10 for C10S SAMs through external compression. This GF surpasses values reported for conjugated wires^55^ and DNA strands,^56^ highlighting the potential of CnS SAMs in mechanosensitive devices.

The strong dependence of GF on the alkyl chain length, corroborated by theoretical simulations, underscores the combined influence of tilt angle and probe penetration on this enhancement. This effect leverages the low Young’s modulus of alkyl chains, enabling significant modulation of electron transport under compression. Conversely, introducing a rigid graphene interlayer with a high Young’s modulus effectively prevents probe penetration. This constraint leads to a GF that is largely independent of the alkyl chain length, remaining relatively constant at approximately 3.5.

These findings pave the way for incorporating CnS SAMs into molecular electronic devices designed for mechanosensitive and mechanocontrollable applications. This includes areas such as touch-sensitive electronic skin and advanced sensor technologies, aligning with the growing demand for flexible and responsive electronic interfaces.^44−46^ Further exploration of these systems can focus on tailoring the mechanical and electrical properties of the SAMs through molecular engineering and interface modifications to optimize their performance in specific applications.

## Experimental Section

### Liquid Phase Exfoliated Graphene (LEG) Preparation Method

Liquid-phase exfoliated graphene (LEG) was prepared by adapting the method outlined by Noveselov et al.^63^ Initially, 10 mg of expandable graphite (500 mesh, Sigma-Aldrich) underwent microwave expansion (800 W, Galanz) for 30 s, resulting in a 4-fold volume increase. This expansion process effectively weakens the π–π stacking interactions between the graphene layers, facilitating exfoliation. The expanded graphite (1 g) was then dispersed in 100 mL of Cyrene (Sigma-Aldrich, concentration of 10 mg/mL) and subjected to bath ultrasonication (40 kHz, 180 W, Skypmen JM-7D) for 14 h. Continuous ice addition to the ultrasonication bath maintained a stable temperature, mitigating potential oxidation and defect formation in the graphene structure. A multistep centrifugation process was employed to size-select the exfoliated graphene flakes (Sunne SN-LSC-4). The dispersion was initially centrifuged at 500 rpm to remove large, unexfoliated particles. The supernatant was then subjected to a second centrifugation step at 2500 rpm, discarding the supernatant containing ultrasmall nanoparticles. The resulting sediment, enriched with LEG flakes, was redispersed in Cyrene at a concentration of 1 mg/mL and centrifuged again at 500 rpm. The supernatant from this final centrifugation step, containing the desired LEG flakes, was carefully filtered through a 300 mesh steel screen, yielding the final LEG dispersion for subsequent use.

### Self-Assembled Monolayers (SAMs) Preparation Method

Template-stripped gold (Au^TS^) substrates were utilized for the formation of self-assembled monolayers (SAMs). AuTS substrates were prepared following the procedure reported by Whitesides et al.^64^ Briefly, a 150 nm gold layer was deposited onto a silicon substrate (0.5 cm × 0.5 cm) via thermal evaporation. A small droplet (∼30 μL) of heat-cure epoxy (Epotek 353nd) was dispensed onto the gold surface by using a syringe needle. A silicon dioxide wafer (0.5 cm × 0.5 cm) was then carefully placed atop the epoxy droplet, forming Si/Au/Glue/SiO_2_ structure. This structure underwent curing at 120 °C for 30 min, ensuring strong adhesion of the epoxy. Subsequently, the structure was cleaved using a surge blade, exposing the Au^TS^ surface (Au/Glue/SiO2). The Au^TS^ substrate exhibits a remarkably smooth surface with a roughness of 0.1 nm (characterized by Bruker MultiMode 8 atomic force microscopy (AFM) in contact mode, SI, Figure S2), approximately 10 times lower than that of thermally evaporated gold, promoting the formation of highly ordered SAMs.

Alkyl thiol self-assembled monolayers (SAMs) were formed on gold thin films (Au^TS^) using a previously reported method.^39^ Briefly, a 10 mM solution of the targe alkyl thiol (Aladdin, purity: C6S 96%, C8S 98%, C10S 99%) was prepared in ethanol (Aladdin, purity >99.8%). Au^TS^ substrates were immersed in the solution and purged with nitrogen for 10 min via syringe to remove dissolved oxygen. SAM growth was carried out over 24 h at room temperature. Following growth, the Au^TS^ substrates were rinsed with methanol, ethanol, and 2-propanol, and then dried under vacuum at 40 °C (<30 Pa) in vacuum oven (JingFei Tech., DZF-6020A) for 1 h to remove residual solvent.

The quality of the alkyl thiol self-assembled monolayers (SAMs) was confirmed using quartz crystal microbalance (QCM) and atomic force microscopy (AFM) nanoscratching, as previously reported. The QCM measurements were conducted on a Au-QCM crystal (5 mm diameter, initial frequency *f*_0_ = 10 MHz, q-Sensor). The QCM resonance frequency was cleaned by ethanol, methanol, and 2-propanol for several times and dried in vacuum chamber overnight. The QCM resonance frequency of the cleaned Au-QCM crystal was recorded using a commercial monitoring system (OpenQCM). After recording, SAMs was deposited onto the Au substrate using the method described above. The difference of frequency before and after SAMs deposition, Δ*f*, was related to the single molecular occupation area, *A*_molecule_, via the Sauerbrey equation:^65^
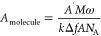

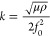
*A*′ is the electrode area, *A* is the crystal area, Δ*f* is the frequency change, *M*_w_ is the molecular weight, *N*_A_ is the Avogadro’s number, μ is the shear modulus of quartz, ρ is the density of quartz, and *f*_0_ is the initial frequency.

The thickness of the self-assembled monolayers (SAMs) was determined using the nanoscratching technique, as described in our previous work.^39,66^ An atomic force microscopy (AFM) probe was employed to scratch a small area (100 nm × 100 nm) in contact mode with a force of 20 nN. Subsequently, a larger area scan was performed using tapping mode over a larger scale (1 μm × 1 μm). The discrepancy between the scratched and nonscratched region is indicative of the film’s thickness.

### LEG/SAMs Preparation and Characterization Method

Prior to use, the liquid exfoliated graphene (LEG) solution was bath-sonicated for 10 min to ensure homogeneity. A gold thin film substrate (Au^TS^) functionalized with a self-assembled monolayer (SAM) was secured on a spin coater under vacuum. Then 100 μL of the LEG dispersion was then evenly deposited onto the rotating Au^TS^ at 100 rpm, ensuring complete surface coverage. Following deposition, the spin speed was increased to 500 rpm for 40 s to facilitate graphene deposition. Finally, the spin coater was accelerated to 2000 rpm for 2 min to remove residual solvent.

### Nanomechanics Characterization Method

Nanomechanical characterization of the SAM and LEG/SAM surfaces was conducted using a Bruker Multi-Mode 8 atomic force microscope (AFM), operating in PeakForce Quantitative Nanomechanics (PF-QNM) mode using a Multi-75G (Budget Sensor, *k* = 3 N/m) probe. Measurements were performed at a frequency of 1.5 kHz and a tapping amplitude of 150 nm. Force spectroscopy data was acquired by collecting force–distance curves at precisely selected points on the SAM and LEG/SAM regions identified from topographic images. The Young’s modulus was then calculated for each force curve using the Derjaguin–Muller–Toporov (DMT) model and averaged over all curves using Nanoscope Analysis software.

### Electric Measurement Method

The electrical characterization was performed by using conductive atomic force microscopy (c-AFM) in contact mode. A platinum-coated AFM probe (ElectriMulti-75G, force constant of 3 N/m) connected to a Keithley 2400 source meter was used to apply a bias voltage, while the sample was grounded to a macroscopic metal plate. The probe was precisely positioned on regions with and without graphene coverage to form probe/SAMs/Au and probe/LEG/SAMs/Au junctions, respectively. A constant tip–sample force was maintained by controlling the deflection error set point. Current–voltage (*I*–*V*) characteristics were acquired by using a National Instruments Data Acquisition (NI-DAQ) card. For each junction type, more than 80 *I*–*V* curves were collected from at least 10 different locations. Differential conductance was calculated by performing a linear regression on every five consecutive data points of the *I*–*V* curves and extracting the slope using a Python script.

### Theoretical Simulation Method

To understand how the insertion of a graphene layer (Gr), affects the magnitude of the tunneling decay factor β, we computed the electrical transport properties of metal/alkyl chain/metal and metal/Gr-alkyl chain/metal junctions. The transport properties of 12 junctions composed of monolayers and bilayers were modeled using a combination of density functional theory (DFT) and quantum transport theory.^64^ These methods have been used over the past two decades to predict the effect on transport properties of a range molecular features, including conformation,^65^ pendent groups,^66^ heteroatoms, and molecular-scale quantum interference. To calculate the electrical transport through monolayers of C6S, C8S, and C10S and bilayers C6S-Gr, C8S-Gr, and C10S-Gr, we modeled junctions formed from the single molecules shown in SI, Figure S6, and the molecular bilayers alkyl chains–graphene sheet (for more details see section 2 in the SI). Furthermore, we calculate the wave function for each of the CnS SAMs (*n* = 6,8,10), the highest occupied molecular orbital (HOMO), lowest unoccupied orbital (LUMO), and their neighbors (i.e., HOMO–1, LUMO+1 etc.), along with their energies are shown in SI, Figures S7, S8, and S9. The optimal binding distances between the electrodes and the different compounds were obtained by calculating their binding energies as a function of distance, as shown in SI, Figures S10–S12. The data are summarized in SI, Table S1.

In this study an external compression was imposed using two different loaded forces: 2 and 10 nN. These load forces and their equivalent tilt angles are shown in SI, Tables S2–S3. The resulting transmission coefficients of the tilt angles are shown in SI, Figures S13–S16.

The results show that the transmission coefficients near the DFT-predicted Fermi energies for probe/CnS/Au are close to HOMO-resonance, whereas for probe/LEG/CnS/Au (Gr-alkyl chains), are close to LUMO resonance (SI, Figure S14 and S16). Then the tunnelling decay β factor has been calculated using eq 2 for the simulations of both with and without graphene sheet (Gr) (Table 2).

**Table 2:** Experiment and Theory (Model 1 and Model 2) Of Determining the Tunnelling Decay β Factor for the Studied Junctions with and without LEG Interlayer

	exp β (nm^–1^)	theo mod 1 β (nm^–1^)	theo mod 2 β (nm^–1^)
CnS-2 nN	8.3	10	12.5
CnS-10 nN	5	7	8.9
LEG/CnS-2 nN	8.1	8.5	NA
LEG/CnS-10 nN	7.8	8.2	NA

We calculated the β factor of the studied junctions as a function of θ for C6S, C8S, and C10S with and without a graphene sheet (Gr), and we compare the experimental β factor against the DFT results as a function of θ as shown in SI, Tables S4–S5. Moreover, SI, Figure S19, shows the results of the transmission coefficients of C6S, C8S, and C10S and bilayers C6S-Gr, C8S-Gr, and C10S-Gr.

Furthermore, the gauge factor was also calculated using eq 5, for the same tilt angles, with and without a Gr sheet as shown in SI, Table S6. Gauge factor theoretical values are compared against the measured ones, as shown in SI, Table S7. However, without the probe penetrating the SAM, the theoretical GFs are lower than the experimental values. Furthermore, the GF is less than one for the shorter chain C6S in both theory and experiment 0.32 and 0.82, respectively. In addition, GFs with the graphene sheets have barely changed 3.4 to 4.4 compared to those without Gr 0.32 to 10.7 (Table 3).

**Table 3:** Experiment and Theory (Models 1 and 2) Of Determining the Gauge Factor for the Studied Junctions with and without LEG Interlayer

	exp GF	theo mod 1 GF	theo mod 2 GF
C6S	0.32	0.82	0.72
C8S	5	4.7	5.5
C10S	10.7	8	12.1
LEG/C6S	3.43	4.9	NA
LEG/C8S	3.8	5.4	NA
LEG/C10S	4.4	5.6	NA

To explain why the GF for the short molecule is less than one and why the GF for different molecular lengths with Gr remains approximately constant, we simulated the effect of the probe penetrating the SAM, because it is reasonable to suppose that, when the tip approaches the SAM with high pressure 10 nN, it penetrates further down toward the substrate (i.e., missing the top anchor group), whereas that is not the case when a layer of Gr sheet is placed between the tip and SAM.

We calculated the tip penetration distances for the three alkyl chains to be 1.51, 3.86, and 7.5 A for C6S, C8S, and C10S, respectively, as shown in SI, Tables S8–S9. The resulting transmission coefficients are recalculated by taking into account the penetration distance above for the three chains and at the two tilt angles 35.5°, 74°. Supporting Information, Figures S20 and S21 indicate that the conductance has increased the more we penetrate, as shown in SI, Table S10. Supporting Information, Figures S22–S23 indicate that the conductance has increased for the longer chains C8S and C10S but decreased for the shorter one C6S, as shown in SI, Table S11. This result is expected as the longer the molecule, the larger the penetration, which leads to a higher conductance. The small penetration distance (1.5 Å) for C6S also explains why the GF is less than one compared to the longer chains. In summary, the GF for chains in the presence of the Gr sheet remains roughly unchanged due to the fact that the graphene sheet prevents the top tip from penetrating the SAM, as shown in SI, Table S12.

## Supporting Information Available

Topographical, nanomechanical and mechano-electric characterization of graphene and SAMs; Frontier orbital wave function of measured molecules; DFT calculated binding energy for all measured junction systems; DFT calculated transmission curve and corresponding β factor for all measured systems; Optimized binding energy for measured junctions; Correlation between measured loading force and tilt angle. Optimized structure of all measured junctions; Experimental and theoretical calculated electric and mechano-electric properties of all measured systems (PDF)
