# Supplementary material for: Enhancing the Pressure-Sensitive Electrical Conductance of Self-Assembled Monolayers
Source: ACS Appl Mater Interfaces. 2024 Nov 19;16(48):66290–300. doi: 10.1021/acsami.4c15796 (PMC11622190; doi:10.1021/acsami.4c15796)
Supplement: Supplementary file 1 — am4c15796_si_001.pdf [file am4c15796_si_001.pdf]

Supporting Information

Enhancing the pressure-sensitive electrical conductance of self-assembled monolayers.

Xintai Wang,[a,b,c,†,*] Asma Alajmi,[a,†] Zhangchenyu Wei,[b,†] Mohammed Alzanbaqi,[a,†] Naixu Wei,[b] Colin Lambert,[a*] and Ali Ismael,[a*]

Table of contents

1.Experiment Section....................2

1.1 Quality of LEG....................2

1.2 Quality of template stripped gold....................3

1.3 YM Distribution for CnS....................4

1.4 Correlation between loading Force, electrode displacement and molecular tilting angle, for probe/SAMs/Au and probe/LEG/SAMs/Au....................4

1.5 correlation between flake size and measured conductance....................5

2.DFT and Transport Calculations....................5

2.1 Frontier orbitals....................5

2.2 Binding energies:....................8

2.2.1 Binding energy of two components....................8

2.2.2 Binding energy of molecules on Au....................9

2.3 The tilt angle (θ)....................10

2.3.1 Transport calculations of (θ)....................12

2.3.2 Tunnelling decay factor as function of (θ)....................14

2.3.3 Gauge factor (GF) as function of (θ)....................17

2.4 Penetration (P)....................18

2.4.1 Transport calculations as a function of (P)....................20

2.4.2 Tunnelling decay factor as a function of (P)....................20

2.4.3 Gauge factor (GF) as a function of (P)....................22

3. References....................25

1.Experiment Section

1.1 Quality of LEG

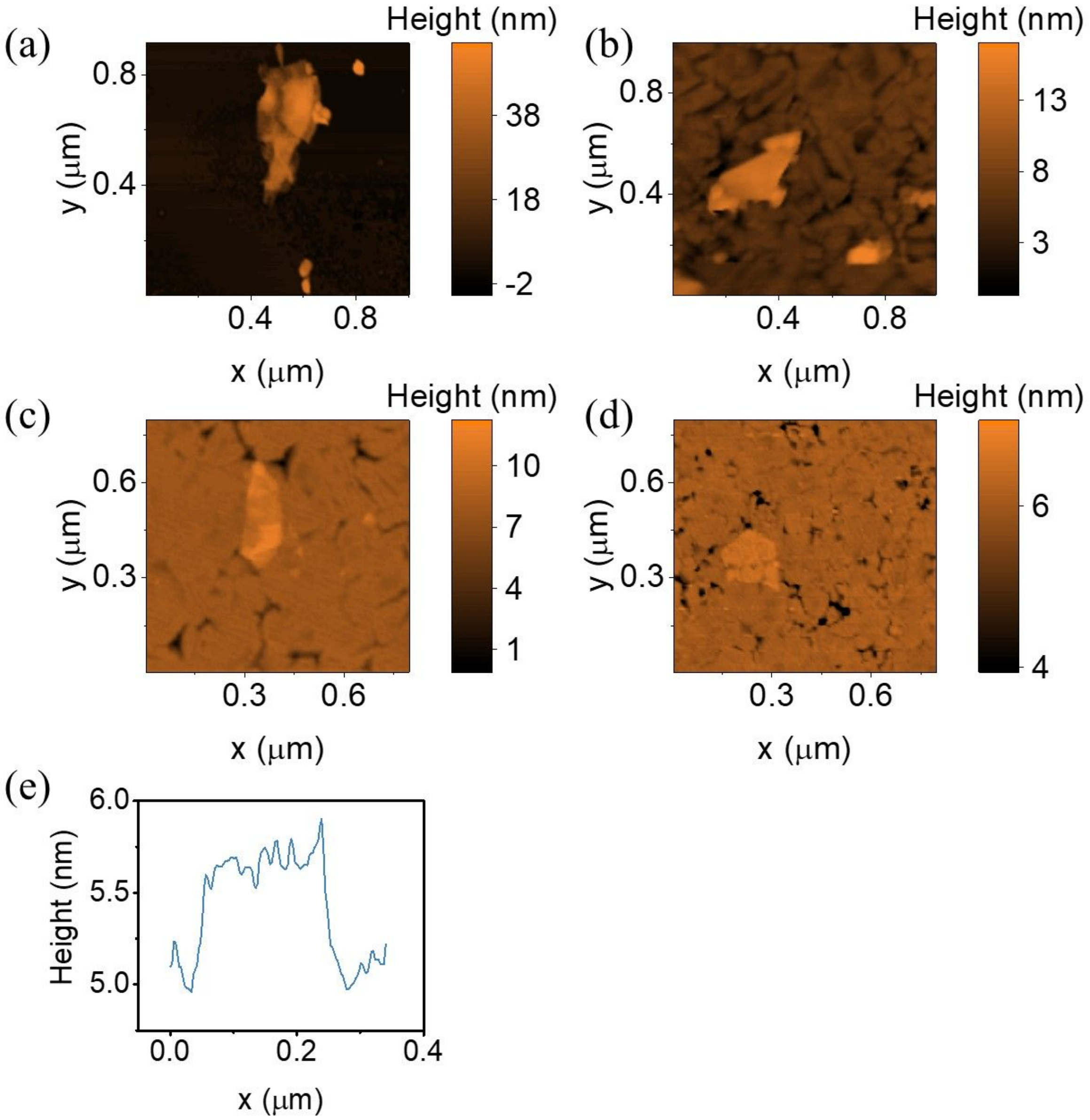

Figure S1: Atomic force microscopy (AFM) characterisation of LEG deposited on SAMs ($C8S$), with LEG exfoliated for 5, 10, 15 and 20 hours (**a-d**), and (**e**) the LEG thickness for (**d**).

1.2 Quality of template stripped gold

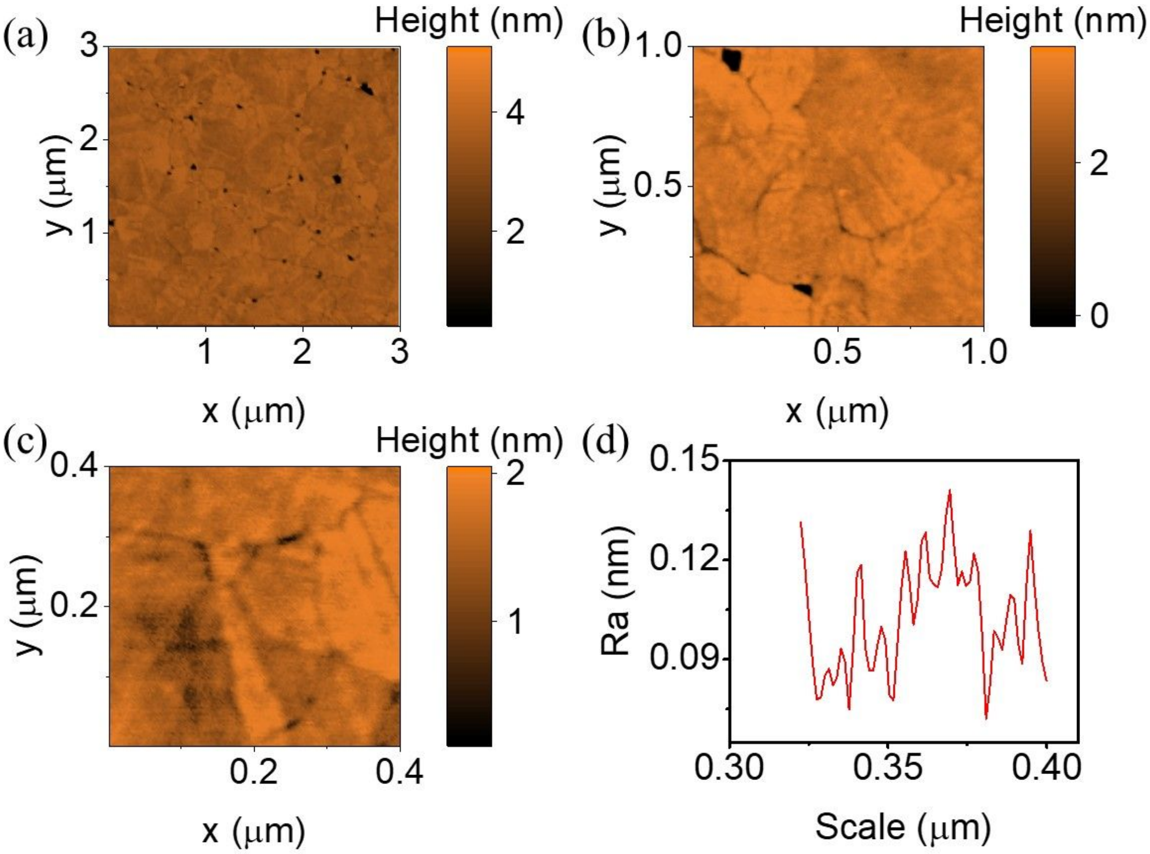

Figure S2: Atomic force microscopy (AFM) characterisation of Au^{TS} substrates. (**a-c**) Representative AFM images of the surface morphology at different magnifications: (**a**) 3 μm, (**b**) 1 μm, and (**c**) 0.4 μm. Images were acquired in contact mode using a force constant of 2 nN. (**d**) Average surface roughness (Ra) values extracted from the corresponding AFM images.

1.3 YM Distribution for CnS

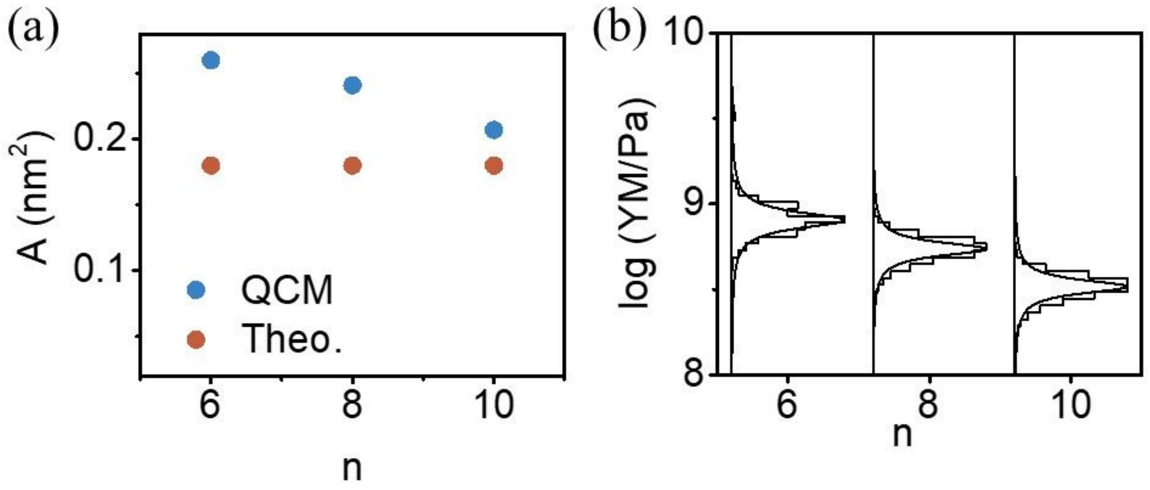

Figure S3: (a) molecular occupation area of CnS/Au SAMs estimated via QCM and comparing with theoretical estimated value. (b) distribution of YM for CnS/Au SAMs.

1.4 Correlation between loading Force, electrode displacement and molecular tilting angle, for probe/SAMs/Au and probe/LEG/SAMs/Au

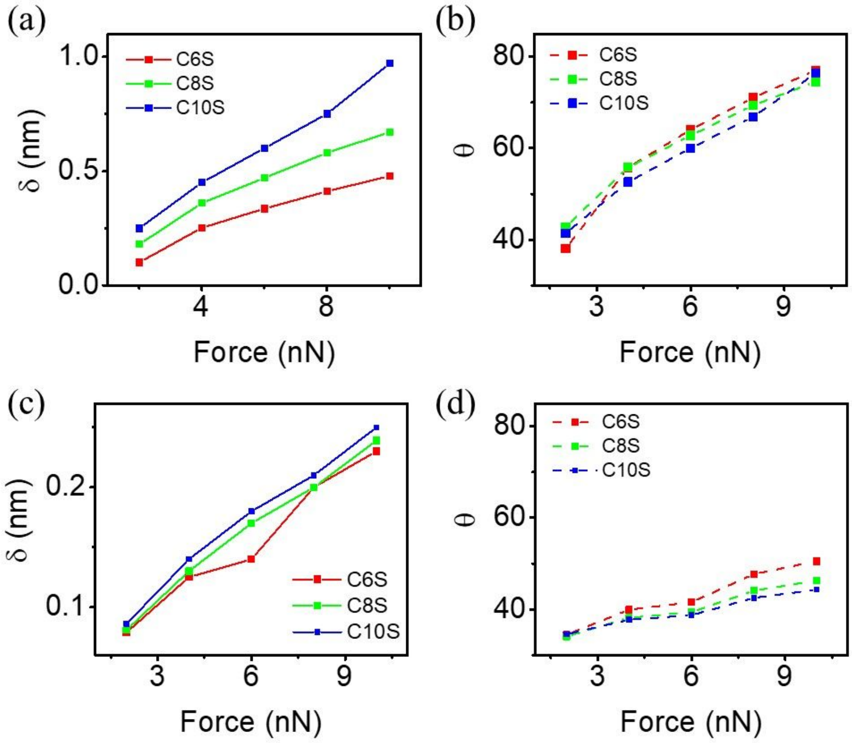

Figure S4: (a) relationship between loading force and electrodes displacement for Probe/SAMs/Au system, and (b) the corresponding tilt angle. (c, d) same as (a, b) but for Probe/LEG/SAMs/Au system.

1.5 correlation between flake size and measured conductance

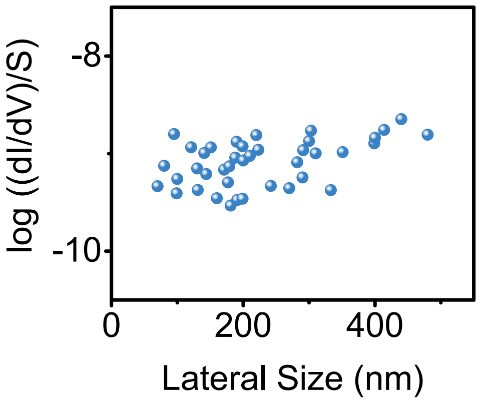

Figure S5: The measured conductance for Probe/LEG/$C8S/Au$ junction, with LEG flake size varying from $\sim 50\ nm\ to \sim 500\ nm.$

2.DFT and Transport Calculations

In this section, geometries, electronic structures and transport properties of all junctions are presented. Using the density functional code SIESTA, the optimum geometries of the isolated molecules were obtained by relaxing the molecules until all forces on the atoms were less than 0.01 eV / Å[5, 6]. A double-zeta plus polarisation orbital basis set, norm-conserving pseudopotentials, an energy cut-off of 250 Rydberg's defining the real space grid were used and the local density approximation (LDA) was chosen as the exchange correlation functional. We also computed results using GGA and found that the resulting transmission functions were comparable with those obtained using LDA[7]. The basic building blocks I-IV of this study are shown in Fig. S6:

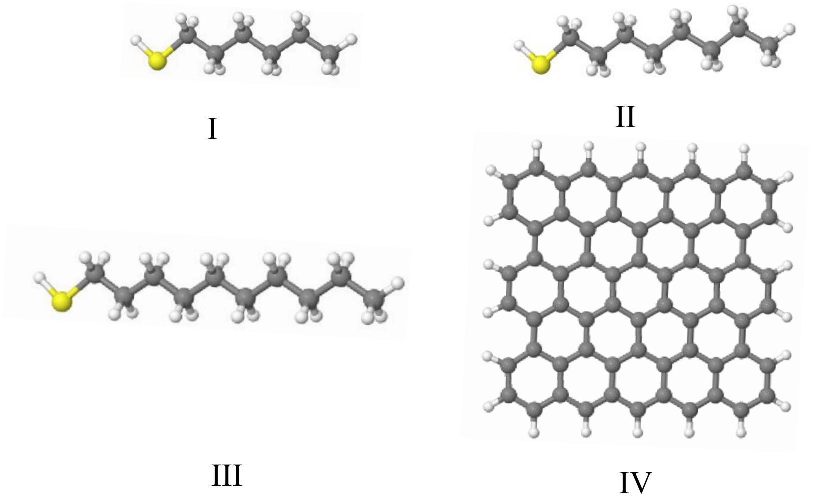

Figure S6: Simulated structures of alkane chain molecules from **I** to **III**, and **IV**: graphene sheet (Gr).

To have a better understanding of the electronic properties of the structures of the studied molecules (Fig. S6). The gas-phase electronic structures of all molecules were investigated to explore the distribution and composition of the frontier molecular orbitals. Plots of the frontier orbitals for the alkane chain molecules are given in Figs. S7-S9 the highest occupied molecular orbitals (HOMO) and lowest unoccupied orbitals (LUMO), (HOMO-1), and (LUMO+1) along with their energies. The blue and red colours represent the negative and positive orbital amplitudes, respectively.

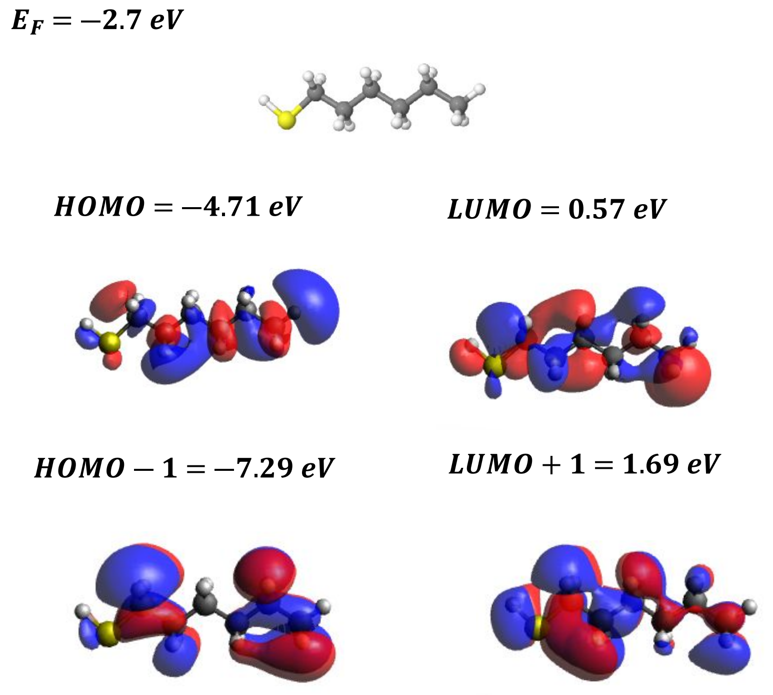

Figure S7: Wave function for $C6S$. **(Top panel):** fully optimised geometry of $C6S$.**(Lower panel):** HOMO, LUMO, HOMO-1, LUMO+1 of $C6S$, along with their energies.

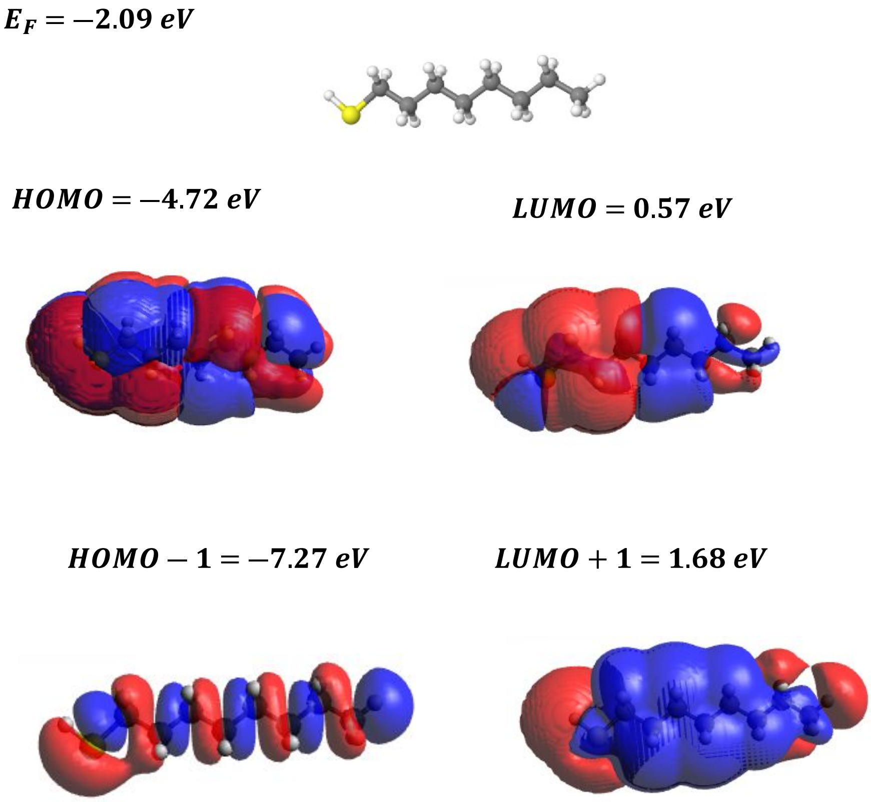

Figure S8: Wave function for $C8S$. **(Top panel):** fully optimised geometry of $C8S$.**(Lower panel):** HOMO, LUMO, HOMO-1, LUMO+1 of $C8S$, along with their energies.

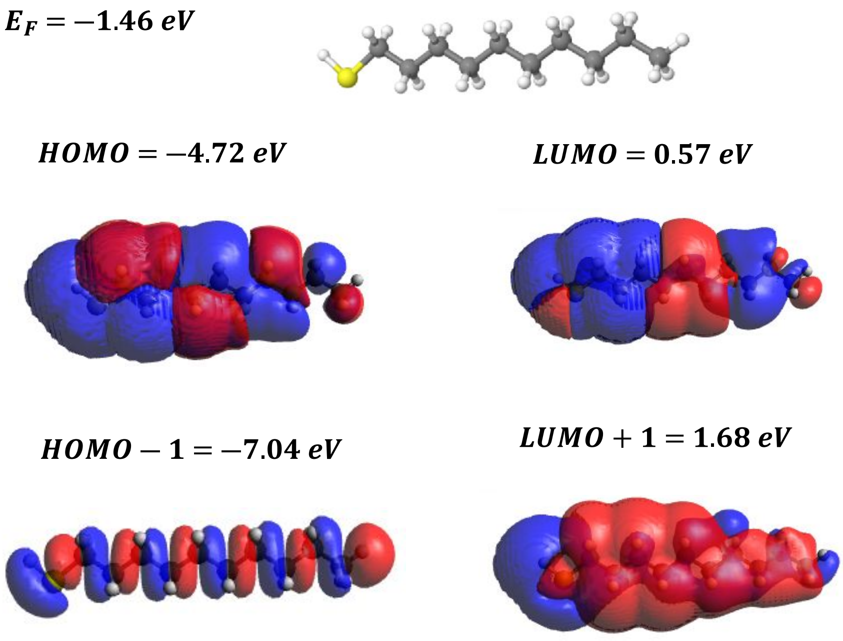

Figure S9: Wave function for $C10S$. **(Top panel):** fully optimised geometry of $C10S$.**(Lower panel):** HOMO, LUMO, HOMO-1, LUMO+1 of $C10S$, along with their energies.

2.2 Binding energies:

To calculate the optimum binding distance between any two components, we used DFT and the counterpoise method, which removes basis set superposition errors (BSSE). The binding distance d is defined as the distance between compound 1 and compound 2. Here, compound 1 is defined as entity A and compound 2 as entity B. The ground state energy of the total system is calculated using SIESTA and is denoted E_{AB}^{AB}. The energy of each entity is then calculated in a fixed basis, which is achieved using ghost atoms in SIESTA[8]. Hence, the energy of the individual 1 in the presence of the fixed basis is defined as E_{A}^{AB} and for the gold as E_{B}^{AB}. The binding energy is then calculated using the following equation:

$$\text{Binding Energy} = E_{AB}^{AB} - E_{A}^{AB} - E_{B}^{AB}$$

Table S1 represents the optimum separation distance and binding energy for three different components C-C, C-Au, and S-Au.

2.2.1 Binding energy of two components

In this section, three binding energies are calculated. $B1$ to find the optimum distance between a terminal C atom and graphene sheet (Gr).

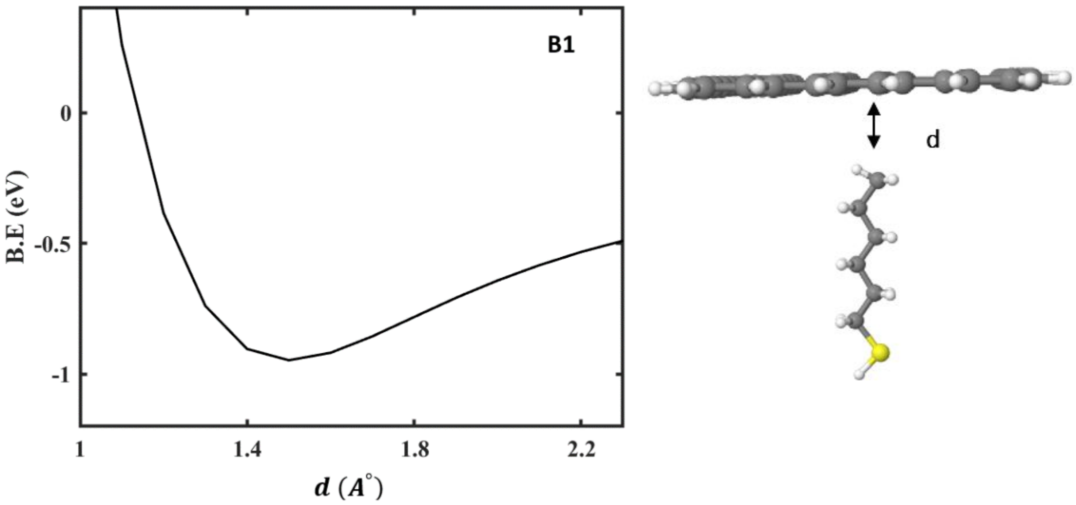

Figure S10: (Left panel): Binding energy as a function of the optimum binding distance d, where d is found to be approximately 1.5 Å, and binding energy $B.E = 0.94\ eV$. **(Right panel):** $B1$ represents molecule binding to a graphene sheet (Gr).

2.2.2 Binding energy of molecules on Au

$B2$ to find the optimum distance d between a Au tip and a graphene sheet and $B3$ between Au, and a thiol anchor, Figs. S11 and S12 show the total energy as a function of distance d. The optimum value of d corresponds to the energy minimum of these curves.

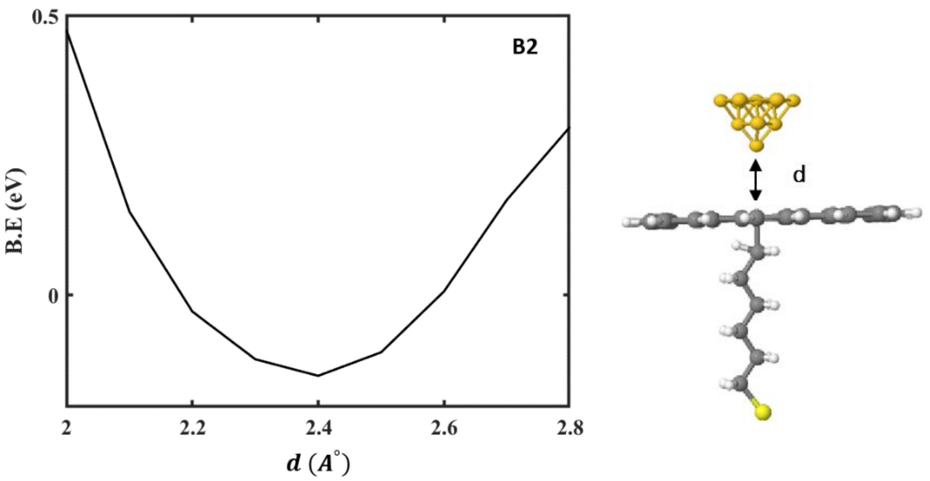

Figure S11: (Left panel): Binding energy as a function of the optimum binding distance d, where d is found to be approximately 2.4 Å, and binding energy $B.E = 0.14\ eV$. **(Right panel):** $B2$ represents molecule binding to an Au lead.

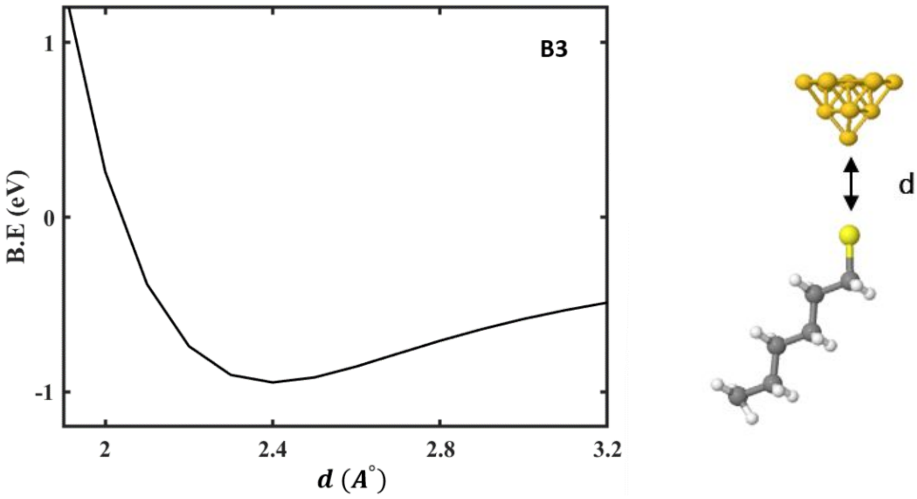

Figure S12: (Left panel): Binding energy as a function of the optimum binding distance d, where d is found to be approximately 2.4 Å, and binding energy $B.E = 0.94\ eV$. **(Right panel):** $B3$ represents molecule binding to an Au lead.

Table S1: Summarises the optimum binding energies and optimum values of d for the three cases ($B1 - B3$)

Compound	$\boldsymbol{B.E\ (eV)}$	$\boldsymbol{d(A^{\circ})}$
$B1$	0.94	1.5
$B2$	0.14	2.4
$B3$	0.94	2.4

2.3 The tilt angle (θ)

In this section, we determine the tilt angle θ of each compound on a gold substrate, which corresponds to the experimentally measured most-probable break-off distance. Table S2 shows each compound for a range of tilt angles. Break-off distance values suggest that the twelve compounds tilt with angle θ ranging 35.5°, 42.2°, and 74.0°. Au/molecules/Au and Au /molecules-Gr/Au[7, 9, 10]. Table S3 shows the optimised structures of the 12 junctions, demonstrating the tilt-angle of the studied molecules.

Table S2: Experimental loaded force in nN unit and equivalent tilt angle (θ)

**Junction**	**Force ($\boldsymbol{nN}$)**	**Equivalent tilt angle ($\boldsymbol{\theta}$)**
$\boldsymbol{Au/C6S/Au}$	2	35.5°
$\boldsymbol{Au/C8S/Au}$		
$\boldsymbol{Au/C10S/Au}$		
$\boldsymbol{Au/C6S/Au}$	10	74.0°
$\boldsymbol{Au/C8S/Au}$		
$\boldsymbol{Au/C10S/Au}$		
$\boldsymbol{Au/C6S - Gr/Au}$	2	35.5°
$\boldsymbol{Au/C8S - Gr/Au}$		
$\boldsymbol{Au/C10S - Gr/Au}$		
$\boldsymbol{Au/C6S - Gr/Au}$	10	42.2°
$\boldsymbol{Au/C8S - Gr/Au}$		
$\boldsymbol{Au/C10S - Gr/Au}$		

Table S3: Optimised structures of the 12 junctions, demonstrating the tilt-angle of the studied molecules (Side-view)

$\boldsymbol{C6S}$ (35.5°)	$\boldsymbol{C6S-Gr}$ (35.5°)	$\boldsymbol{C6S}$ (74°)	$\boldsymbol{C6S-Gr}$ (42.2°)
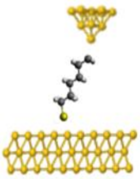	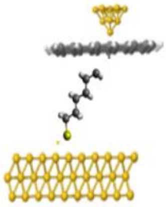	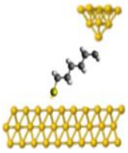	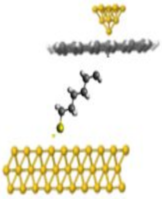
$\boldsymbol{C8S}$ (35.5°)	$\boldsymbol{C8S-Gr}$ (35.5°)	$\boldsymbol{C8S}$ (74°)	$\boldsymbol{C8S-Gr}$ (42.2°)
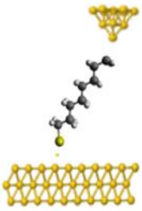	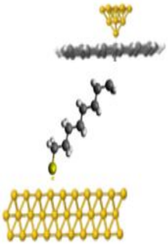	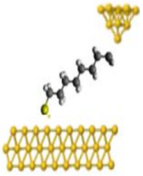	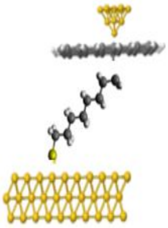
$\boldsymbol{C10S}$ (35.5°)	$\boldsymbol{C10S-Gr}$ (35.5°)	$\boldsymbol{C10S}$ (74°)	$\boldsymbol{C10S-Gr}$ (42.2°)
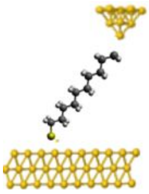	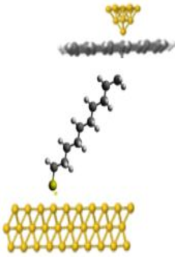	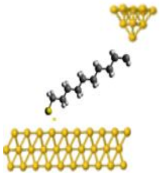	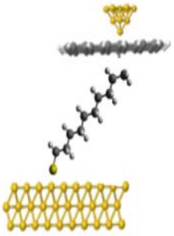

2.3.1 Transport calculations of ($\boldsymbol{\theta}$)

The transmission coefficient curves $T(E)$, obtained using the Gollum transport code, were calculated for the twelve junctions based on the tilt angles (θ) in Table S3, as shown in the Figs. S13-16. The Fermi level of the electrodes in the systems without graphene were $0\ eV$. However, in the systems with graphene, the Fermi level of the electrodes is shifted to $-0.14\ eV$.

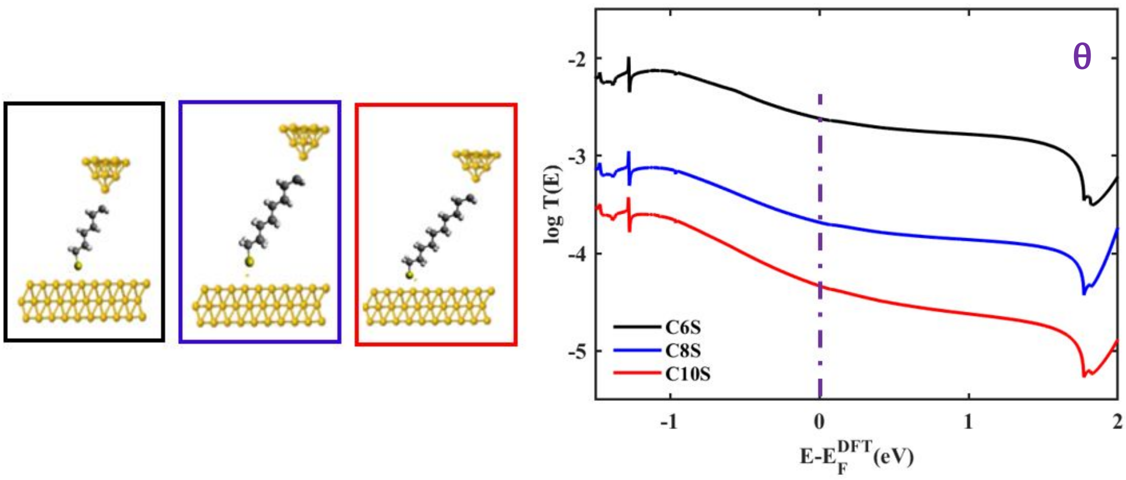

Figure 13: **(Left panel):** Schematic illustrations of molecular junctions for $Au/C6S/Au$, $Au/C8S/Au$ and $Au/C10S/Au$, at θ =35.5°. **(Right panel):** Transmission coefficient $T(E)$ of different junctions, against electron energy E, at θ =35.5° and ($E_F = 0$).

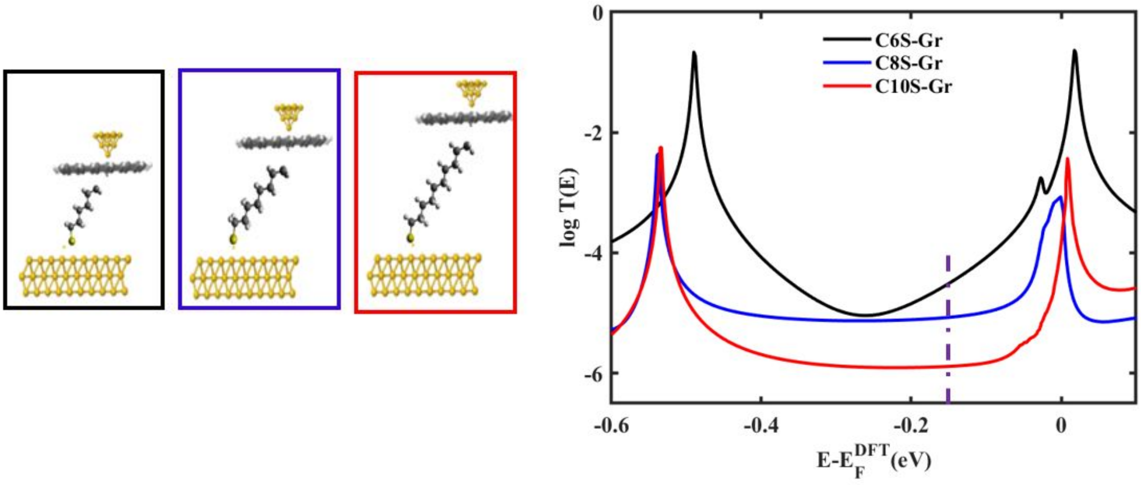

Figure S14: **(Left panel):** Schematic illustrations of molecular junctions for $Au/C6S-Gr/Au$, $Au/C8S-Gr/Au$ and $Au/C10S-Gr/Au$, at θ =35.5°. **(Right panel):** Transmission coefficient $T(E)$ of different junctions, against electron energy E, at θ=35.5° and ($E_F = -0.14$).

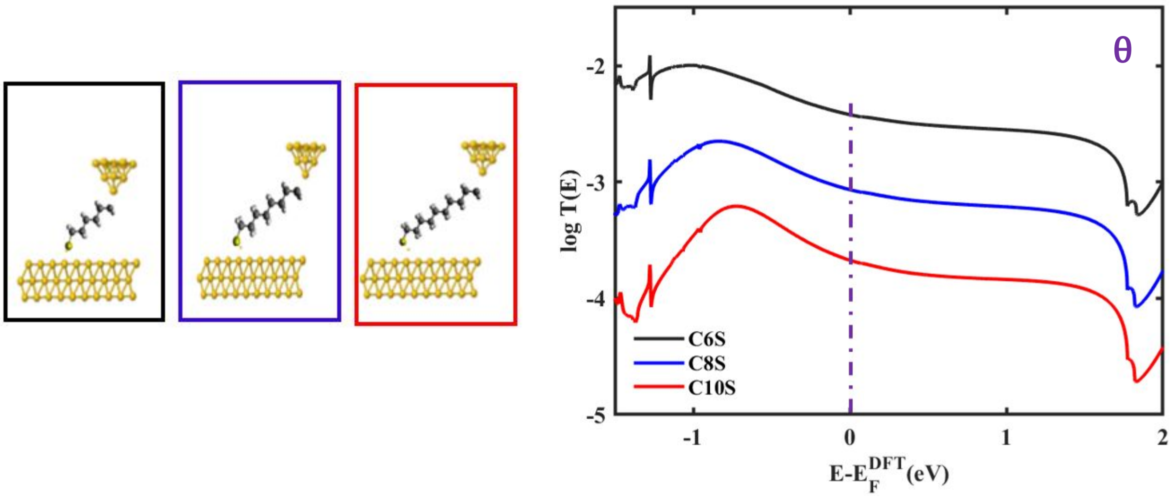

Figure S15: **(Left panel):** Schematic illustrations of molecular junctions for $Au/C6S/Au$, $Au/C8S/Au$ and $Au/C10S/Au$**,** at θ =74°. **(Right panel):** Transmission coefficient $T(E)$ of different junctions, against electron energy E, at θ =74°and ($E_F = 0$).

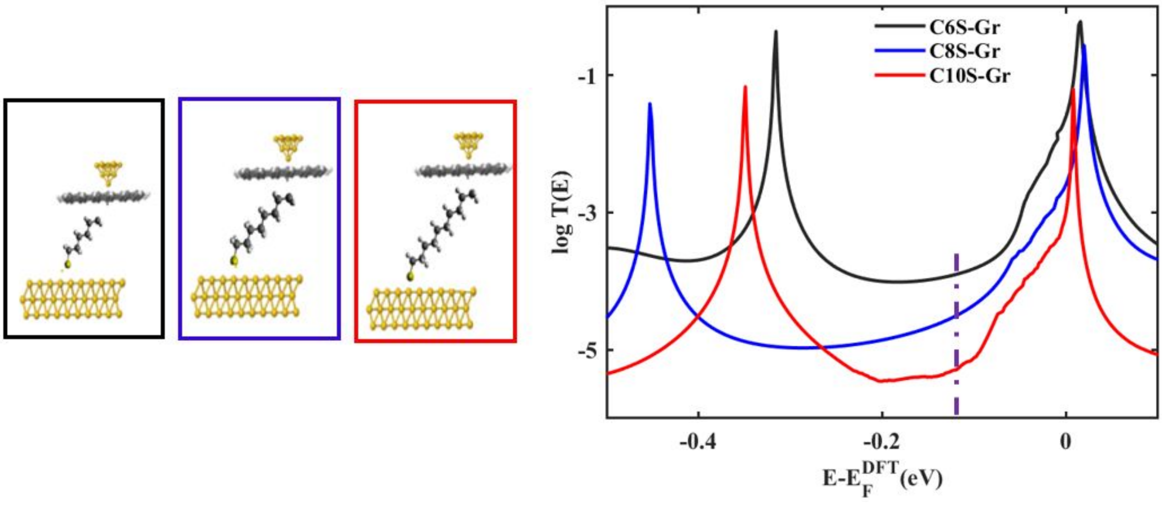

Figure S16: **(Left panel):** Schematic illustrations of molecular junctions for $Au/C6S - Gr/Au$, $Au/C8S - Gr/Au$ and $Au/C10S - Gr/Au$, at θ =42.2°. **(Right panel):** Transmission coefficient $T(E)$ of different junctions, against electron energy E, at θ =42.2°and ($E_F = -0.14$).

2.3.2 Tunnelling decay factor as function of ($\boldsymbol{\theta}$)

To calculate the tunnelling decay beta factor, we use:

$$\ln(G) = \ln(G_0) - \beta d$$

where G is the conductance, d represents the thickness of the layer, G_0 is the initial conductance and β: is the decay constant. This equation is equivalent to

$$\beta = {-\ln\left(\frac{G}{G_0}\right)} \Big/ {d}$$

Table S4 shows $\beta -$ factor of the studied junctions as a function of θ.The β value for the molecules/probe junction $C6S$, $C8S$, and $C10S$ was measured to be $10\, nm^{-1}$ at an angle of 35.5°. Additionally, at an angle of 74°, the β value was found to be 7.0 nm^{-1}. The change in β values for the molecules-Graphene/probe junction ($C6S$, $C8S$, and $C10S$) was measured to be 8.5 nm^{-1} at an angle of 35.5° and 8.2 nm^{-1} at an angle of 42° (Table S4 and Fig. 19(a)). Additionally, these changes are further detailed in (Table S4 and Fig. 19(b))[11-13].

Table S4: Beta factor of the studied junctions as a function of θ

Molecules	Graphene	DFT		
		$\boldsymbol{\theta}$	$\boldsymbol{T(E)}$	$\boldsymbol{\beta}$ ($\boldsymbol{nm^{-1}}$)
$\boldsymbol{C6S}$	No	35.5°	$2.3E-3$	10
$\boldsymbol{C8S}$			$2.0E-4$	
$\boldsymbol{C10S}$			$4.6E-5$	
$\boldsymbol{C6S}$		74°	$3.7E-3$	7.0
$\boldsymbol{C8S}$			$8.6E-4$	
$\boldsymbol{C10S}$			$2.1E-4$	
$\boldsymbol{C6S}$	Yes	35.5°	$3.7E-5$	8.5
$\boldsymbol{C8S}$			$8.7E-6$	
$\boldsymbol{C10S}$			$1.3E-6$	
$\boldsymbol{C6S}$		42.2°	$1.09E-4$	8.2
$\boldsymbol{C8S}$			$2.3E-5$	
$\boldsymbol{C10S}$			$4.1E-6$	

Table S5 shows the $\beta -$ factor versus the DFT results as a function of θ. For comparison, the same study was conducted on the molecules/probe junction. At a low angle of 35.5°, the β value was 10 nm^{-1}. However, at a high angle of 74°, the β value decreased to 7.0 nm^{-1}.

Table S5: Experimental beta factor against the DFT results as a function of θ

Molecules	Graphene	AFM			DFT		
		Force (nN)	G/G_0	β (nm^{-1})	θ	$T(E)$	β (nm^{-1})
$C6S$	No	2	7.2E-5	8.3	35.5°	$2.3E-3$	10
$C8S$			6.5E-6			$2.0E-4$	
$C10S$			6.6E-7			$4.6E-5$	
$C6S$		10	8.8E-5	5	74°	$3.7E-3$	7.0
$C8S$			2.8E-5			$8.6E-4$	
$C10S$			5.3E-6			$2.1E-4$	
$C6S$	Yes	2	5.3E-5	8.1	35.5°	$3.7E-5$	8.5
$C8S$			9.6E-6			$8.7E-6$	
$C10S$			9.5E-7			$1.3E-6$	
$C6S$		10	7.3E-5	7.8	42.2°	$1.09E-4$	8.2
$C8S$			1.3E-5			$2.3E-5$	
$C10S$			2.7E-6			$4.1E-6$	

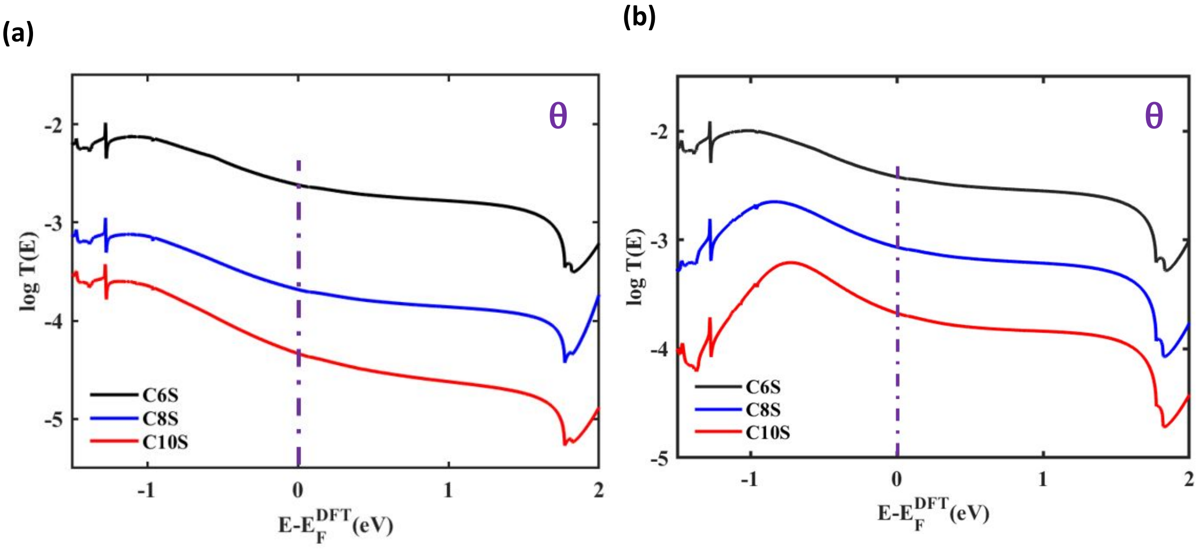

Figure 17: (a) Conductance of junctions without Graphene $C6S$, $C8S$ and $C10S$ ($\beta = 10$). **(b)** Conductance of $C6S$, $C8S$ and $C10S$ ($\beta = 7.0$).

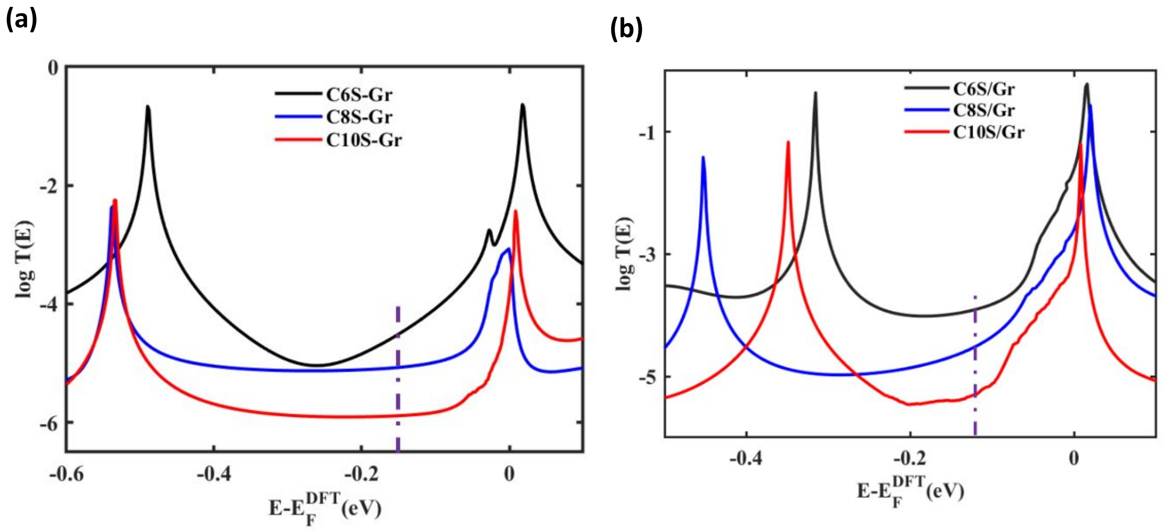

Figure 18: **(a)** Conductance of $C6S-Gr$, $C8S-Gr$ and $C10S-Gr$ ($\beta=8.5$) **(b)** Conductance of $C6S-Gr$, $C8S-Gr$ and $C10S-Gr$ ($\beta=8.2$).

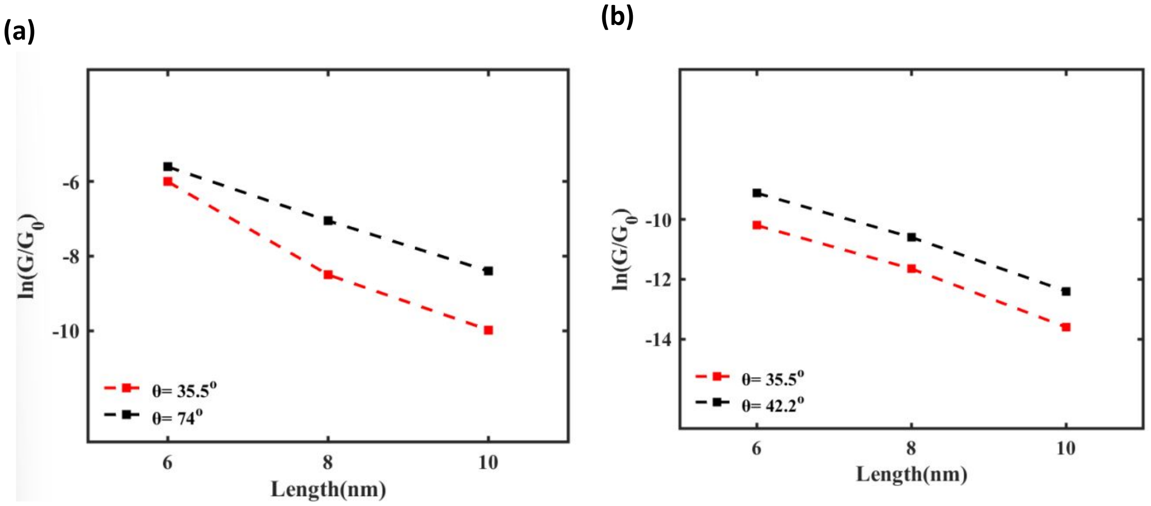

Figure 19: DFT simulations of **(a)** Beta factor of molecules $C6S$, $C8S$, and $C10S$. **(b)** Beta factor of molecules-Graphene.

2.3.3 Gauge factor ($\boldsymbol{GF}$) as function of ($\boldsymbol{\theta}$)

The gauge factor (GF) is a measure of sensitivity in molecular junctions, particularly in the context of strain or deformation. It plays a significant role in understanding the charge transport mechanisms within these junctions. In molecular electronics, this factor helps in the quantification of how electronic properties of molecules change under mechanical deformation or different alignment configurations.

$$GF = (\frac{\Delta G}{G}) \div (\frac{\Delta L}{L})$$

where: ΔG: is the change in G, G is the initial value of the property, ΔL is the change in the film thickness and L is the initial film thickness, given by:

$$L = length\ of\ molecule\ \times \sin{(90^{\circ} - \theta)}$$

θ: is the initial tilt angle.

The GF for the molecules/probe junction was calculated for various molecules. For C6S, the GF was found to be 0.82, while for $C8S$ and $C10S$, it was found to be 4.7 and 8.0, respectively. When considering penetration, the G-factor for $C6S$ decreased to 0.72. In contrast, the GF for $C8S$ and $C10S$ increased to 5.9, and 10.3, respectively.

In contrast, the GF for molecules-Graphene/probe junctions tend to be more stable due to the rigidity and effective load distribution properties of graphene. For $C6S - Gr$, $C8S - Gr$, and $C10S - Gr$, the GFs were calculated to be 4.8, 5.4, and 5.6, respectively, indicating less variability under mechanical stress compared to their non-graphene counterparts (Table S6)[14]. Table S7 shows the G- factor comparison as a function of the tilt-angle θ.

Table S6: GF as a function of the tilt-angle θ for the studied junctions

Molecules	Graphene	DFT		
		θ	$T(E)$	GF
$C6S$	No	35.5°	$2.3E-3$	0.82
		74°	$3.7E-3$	
$C8S$		35.5°	$2.0E-4$	4.7
		74°	$8.6E-4$	
$C10S$		35.5°	$4.6E-5$	8.0
		74°	$2.1E-4$	
$C6S$	Yes	35.5°	$3.7E-5$	4.8
		42.2°	$1.09E-4$	
$C8S$		35.5°	$8.7E-6$	5.4
		42.2°	$2.3E-5$	
$C10S$		35.5°	$1.3E-6$	5.6
		42.2°	$4.1E-6$	

Table S7: G- factor comparison as a function of the tilt-angle θ

Molecules	Graphene	AFM			DFT		
		Force	$G/G0$	GF	θ	$T(E)$	GF
$C6S$	No	$2nN$	7.2E-05	0.32	35.5°	$2.3E-3$	0.82
		$10nN$	8.8E-05		74°	$3.7E-3$	
$C8S$		$2nN$	6.5E-06	5	35.5°	$2.0E-4$	4.7
		$10nN$	2.8E-05		74°	$8.6E-4$	
$C10S$		$2nN$	6.6E-07	10.7	35.5°	$4.6E-5$	8.0
		$10nN$	5.3E-06		74°	$2.1E-4$	
$C6S$	Yes	$2nN$	5.3E-05	3.43	35.5°	$3.7E-5$	4.8
		$10nN$	7.3E-05		42.2°	$1.09E-4$	
$C8S$		$2nN$	9.6E-06	3.8	35.5°	$8.7E-6$	5.4
		$10nN$	1.3E-05		42.2°	$2.3E-5$	
$C10S$		$2nN$	9.5E-7	4.4	35.5°	$1.3E-6$	5.6
		$10nN$	1.7E-6		42.2°	$4.1E-6$	

2.4 Penetration ($\boldsymbol{P}$)

In this section, the penetration (P) describes how deep the top tip penetrates towards the Au substrate. In the previous section, we demonstrated how the tilt angle determines the beta and gauge factors; however, the results are not entirely conclusive. Table S8 presents the penetration (P) values for each compound under different tilt angle conditions. The loaded force values suggest that for the six studied, compounds penetration occurs at an angle θ of

35.5°: $C6S$ penetrates to 1.51 Å, $C8S$ to 3.86 Å, and $C10S$ to 7.5 Å. At an angle of 74.0°, the penetration values are: $C6S$: 2.0 Å, $C8S$: 4.1 Å, and $C10S$: 8.1 Å as shown in Table S8.

It is important to note that, experimentally, the penetration (P) could not be studied for Au /molecules-Gr/Au junctions because the graphene layer (Gr) effectively shields the molecules from penetration (P). Table S9 shows the optimised structures of the six junctions, demonstrating the penetration (P) of the molecules.

Table S8: Penetration (P) values at two different tilt angles

Junction	Penetration (P) at $\theta = 35.5\ A^{\circ}$	Penetration (P) at $\theta = 74.0\ A^{\circ}$
$\boldsymbol{Au/C6S/Au}$	$1.51A^{\circ}$	$2.0A^{\circ}$
$\boldsymbol{Au/C8S/Au}$	$3.86A^{\circ}$	$4.1A^{\circ}$
$\boldsymbol{Au/C10S/Au}$	$7.5A^{\circ}$	$8.1A^{\circ}$

Table S9: Optimised structures of the 6 junctions, demonstrating the penetration (P) of the molecules (Side-view)

$\boldsymbol{C6S}$ (35.5°)	$\boldsymbol{C6S}$ (74°)	$\boldsymbol{C8S}$ (35.5°)	$\boldsymbol{C8S}$ (74°)	$\boldsymbol{C10S}$ (35.5°)	$\boldsymbol{C10S}$ (74°)
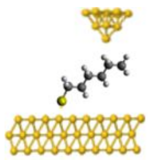	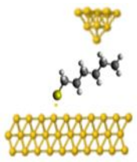	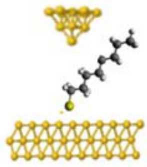	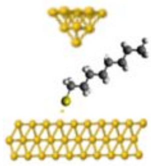	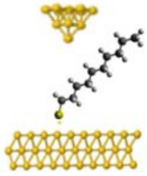	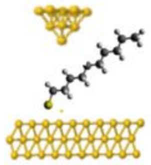

2.4.1 Transport calculations as a function of (P)

The transmission coefficient curves $T(E)$ for the six junctions, calculated using the Gollum transport code, are presented in Figs. S20-21 and correspond to the penetration (P) values listed in Table S8. The systems without graphene have electrodes with a Fermi level set at 0 eV.

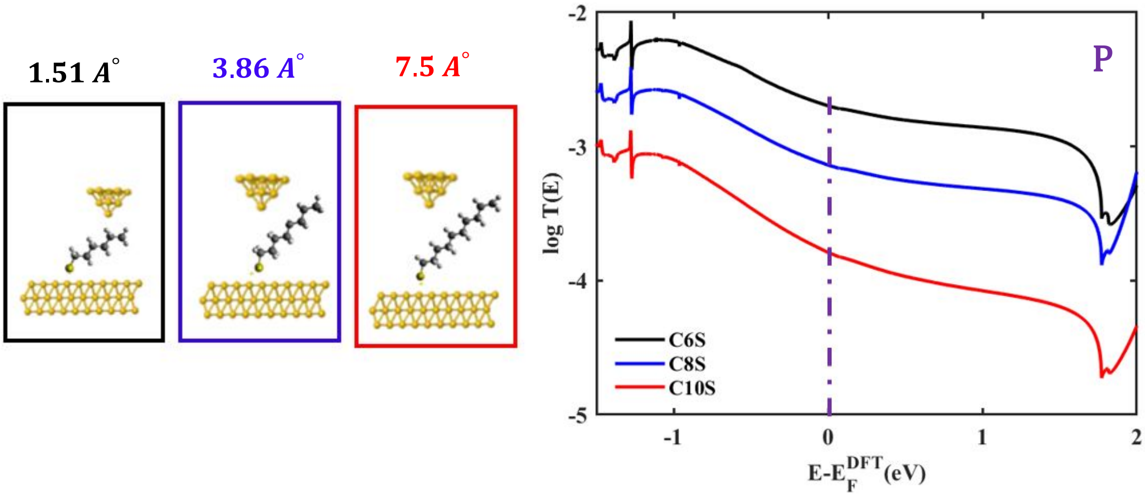

Figure 20: (Left panel): Schematic illustrations of molecular junctions at different penetration (P) distances for $Au/C6S/Au$, $Au/C8S/Au$ and $Au/C10S/Au$. **(Right panel):** Transmission coefficient $T(E)$ of different junctions, against electron energy E. $(\beta = 12.5)(E_F = 0)$.

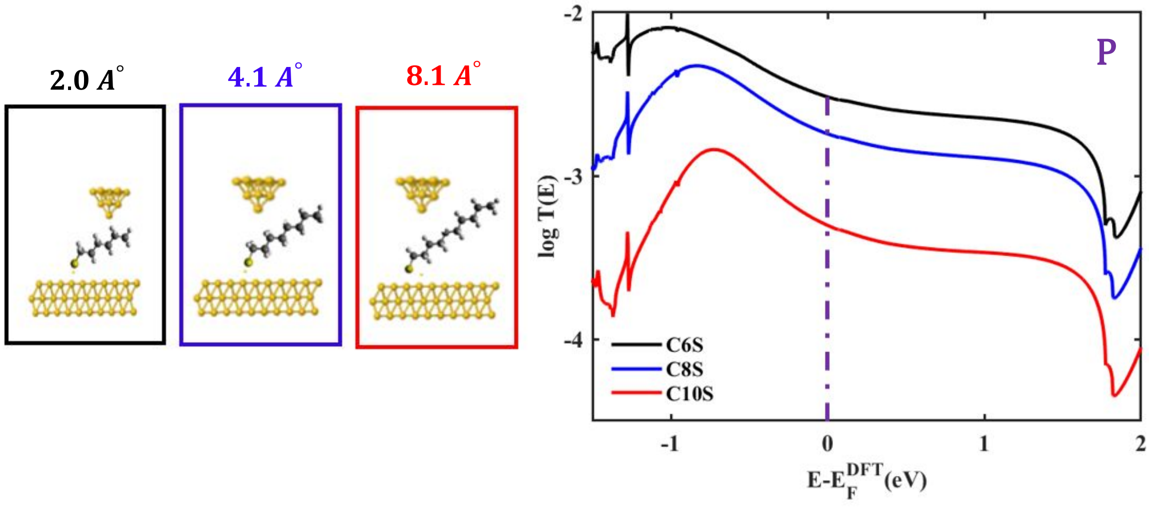

Figure S21: (Left panel): Schematic illustrations of molecular junctions at different penetration (P) distances for $Au/C6S/Au$, $Au/C8S/Au$ and $Au/C10S/Au$. **(Right panel):** Transmission coefficient $T(E)$ of different junctions, against electron energy E. $(\beta = 8.9)(E_F = 0)$.

The β value for the molecules/probe junction ($C6S$, $C8S$, and $C10S$) increased to $12.5\ nm^{-1}$ at a tilt angle of 35.5°, and similarly, it increased to $8.9\ nm^{-1}$ at a tilt angle of 74°. In contrast, the β values for the molecules-Graphene/probe junction ($C6S-Gr$, $C8S-Gr$, and $C10S-Gr$) remained constant (Table S10 and Fig. 23).

Table S10: Beta factor simulations for both θ and P

**Molecules**	**Graphene**	**DFT**					
		$\boldsymbol{\theta}$	$\boldsymbol{T(E)}$	$\boldsymbol{\beta}$ $\boldsymbol{(nm^{-1})}$	$\boldsymbol{P}$	$\boldsymbol{T(E)}$	$\boldsymbol{\beta}$ $\boldsymbol{(nm^{-1})}$
$\boldsymbol{C6S}$	No	35.5°	$2.3E-3$	10	$1.51\ A^{\circ}$	$2.01E-3$	12.5
$\boldsymbol{C8S}$			$2.0E-4$		$3.86\ A^{\circ}$	$7.26E-4$	
$\boldsymbol{C10S}$			$4.6E-5$		$7.5\ A^{\circ}$	$1.62E-4$	
$\boldsymbol{C6S}$		74°	$3.7E-3$	7.0	$2.0\ A^{\circ}$	$3.03E-3$	8.9
$\boldsymbol{C8S}$			$8.6E-4$		$4.1\ A^{\circ}$	$1.80E-3$	
$\boldsymbol{C10S}$			$2.1E-4$		$8.1\ A^{\circ}$	$4.96E-4$	
$\boldsymbol{C6S}$	Yes	35.5°	$3.7E-5$	8.5		$3.7E-5$	8.5
$\boldsymbol{C8S}$			$8.7E-6$			$8.7E-6$	
$\boldsymbol{C10S}$			$1.3E-6$			$1.3E-6$	
$\boldsymbol{C6S}$		42.2°	$1.09E-4$	8.2		$1.09E-4$	8.2
$\boldsymbol{C8S}$			$2.3E-5$			$2.3E-5$	
$\boldsymbol{C10S}$			$4.1E-6$			$4.1E-6$	

We compared the conductance dependence on the tilt angle and penetration. The deformation of molecules under high pressure further modifies the tunnelling regime, leading to a notable reduction in the tunnelling decay factor. During penetration at varying distances, the β value increased from 10 nm^{-1} to $12.5\ nm^{-1}$, and from $7.0\ nm^{-1}$ to $8.9\ nm^{-1}$. In contrast, for the molecules-Graphene/probe junction, the rigid graphene distributes the loading pressure, preventing cross-talk and deformation.

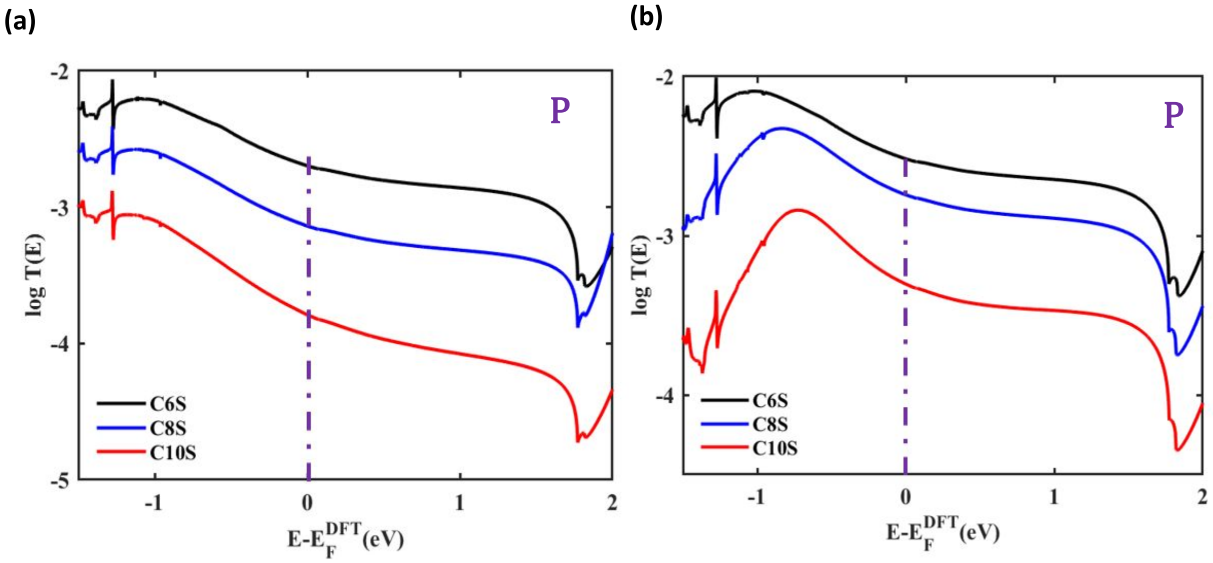

Figure 22: (a) Conductance of penetration junctions without Graphene $C6S$, $C8S$ and $C10S$ ($\beta = 12.5$). **(b)** Conductance of $C6S$, $C8S$ and $C10S$ ($\beta = 8.9$).

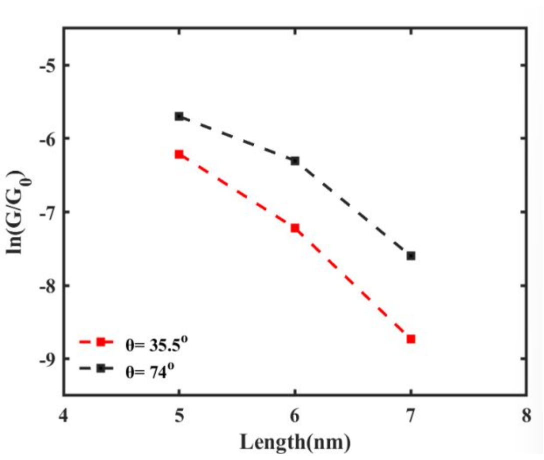

Figure 23: Beta factor as a function of penetration for $C6S$, $C8S$, and $C10S$.

2.4.3 Gauge factor ($\boldsymbol{GF}$) as a function of (P)

Table S11 shows the DFT simulations of G- factor as function of the tilt-angle and penetration for the studied junctions. The GF, versus penetration, for the molecules/probe junction was measured for various molecules. For $C6S$, the G-factor decreased to 0.72. In contrast, the G-factor for $C8S$ increased to 5.9, and for $C10S$, it increased to 10.3. Table S12 shows the G-

factor theory versus experiment results. And Table S13 shows the GF of all measured systems, calculated via equation: $GF = |(\frac{\Delta R}{R})/(\frac{\delta}{L})|$.

Table S11: DFT simulations of G- factor as function of the tilt-angle and penetration θ *and* P for the studied junctions

Molecules	Graphene	DFT					
		$\boldsymbol{\theta}$	$\boldsymbol{T(E)}$	$\boldsymbol{GF}$	$\boldsymbol{P}$	$\boldsymbol{T(E)}$	$\boldsymbol{GF}$
$\boldsymbol{C6S}$	No	35.5°	$2.3E-3$	0.82	$1.51\ A^{\circ}$	$2.01E-3$	0.72
		74°	$3.7E-3$		$2.0\ A^{\circ}$	$3.03E-3$	
$\boldsymbol{C8S}$		35.5°	$2.0E-4$	4.7	$3.86A^{\circ}$	$7.26E-4$	5.9
		74°	$8.6E-4$		$4.1\ A^{\circ}$	$1.80E-3$	
$\boldsymbol{C10S}$		35.5°	$4.6E-5$	8.0	$7.5\ A^{\circ}$	$1.62E-4$	10.3
		74°	$2.1E-4$		$8.1\ A^{\circ}$	$4.96E-4$	
$\boldsymbol{C6S}$	Yes	35.5°	$3.7E-5$	4.8		$3.7E-5$	4.8
		42.2°	$1.09E-4$			$1.09E-4$	
$\boldsymbol{C8S}$		35.5°	$8.7E-6$	5.4		$8.7E-6$	5.4
		42.2°	$2.3E-5$			$2.3E-5$	
$\boldsymbol{C10S}$		35.5°	$1.3E-6$	5.6		$1.3E-6$	5.6
		42.2°	$4.1E-6$			$4.1E-6$	

Table S12: G- factor theory and experiment comparison

Table S13: measured calculated GF of all systems, via equation: $GF = |(\frac{\Delta R}{R})/(\frac{\delta}{L})|$

Junction	$\Delta\theta$ (°)	δ (nm)	GF (ΔR/ R)
**Probe/$C6S$/Au**	38.1	0.38	0.252
**Probe/$C8S$/Au**	32.2	0.49	3.92
**Probe/$C10S$/Au**	34.5	0.74	6.58
**Probe/LEG/$C6S$/Au**	9.2	0.13	1.96
**Probe/LEG/$C8S$/Au**	12.2	0.14	2.38
**Probe/LEG/$C10S$/Au**	16.1	0.19	3.92

Molecules	Graphene	STM			DFT		
		Force	$G/G0$	$G - Factor$	P	$T(E)$	$G - Factor$
$C6S$	No	$2nN$	7.2E-05	0.32	$1.51\ A^{\circ}$	$2.01E-3$	0.72
		$10nN$	8.8E-05		$2.0\ A^{\circ}$	$3.03E-3$	
$C8S$		$2nN$	6.5E-06	5	$3.86A^{\circ}$	$7.26E-4$	5.9
		$10nN$	2.8E-05		$4.1\ A^{\circ}$	$1.80E-3$	
$C10S$		$2nN$	6.6E-07	10.7	$7.5\ A^{\circ}$	$1.62E-4$	10.3
		$10nN$	5.3E-06		$8.1\ A^{\circ}$	$4.96E-4$	
$C6S$	Yes	$2nN$	5.3E-05	3.43		$3.7E-5$	4.8
		$10nN$	7.3E-05			$1.09E-4$	
$C8S$		$2nN$	9.6E-06	3.8		$8.7E-6$	5.4
		$10nN$	1.3E-05			$2.3E-5$	
$C10S$		$2nN$	4.5E-11	4.4		$1.3E-6$	5.6
		$10nN$	6.7E-11			$4.1E-6$	

3. References

1. Pan, K.W., et al., *Sustainable production of highly conductive multilayer graphene ink for wireless connectivity and IoT applications.* Nature Communications, 2018. **9**.
2. Weiss, E.A., et al., *Si/SiO2-Templated formation of ultraflat metal surfaces on glass, polymer, and solder supports: Their use as substrates for self-assembled monolayers.* Langmuir, 2007. **23**(19): p. 9686-9694.
3. Dappe, Y.J., *Attenuation Factors in Molecular Electronics: Some Theoretical Concepts.* Applied Sciences-Basel, 2020. **10**(18).
4. Wang, X.T., et al., *Determination of electric and thermoelectric properties of molecular junctions by AFM in peak force tapping mode.* Nanotechnology, 2023. **34**(38): p. 385704.
5. Soler, J.M., et al., *The SIESTA method for<i>ab initio</i>order-<i>N</i>materials simulation.* Journal of Physics: Condensed Matter, 2002. **14**(11): p. 2745-2779.
6. Artacho, E., et al., *The SIESTA method; developments and applicability.* Journal of Physics: Condensed Matter, 2008. **20**(6): p. 064208.
7. Herrer, L., et al., *Single molecule <i>vs</i>. large area design of molecular electronic devices incorporating an efficient 2-aminepyridine double anchoring group.* Nanoscale, 2019. **11**(34): p. 15871-15880.
8. Alshammari, M., et al., *Orientational control of molecular scale thermoelectricity.* Nanoscale Advances, 2022. **4**(21): p. 4635-4638.
9. Milan, D.C., et al., *The single-molecule electrical conductance of a rotaxane-hexayne supramolecular assembly.* Nanoscale, 2017. **9**(1): p. 355-361.
10. Davidson, R.J., et al., *Conductance of 'bare-bones' tripodal molecular wires.* RSC Advances, 2018. **8**(42): p. 23585-23590.
11. Engelkes, V.B., J.M. Beebe, and C.D. Frisbie, *Analysis of the Causes of Variance in Resistance Measurements on Metal−Molecule−Metal Junctions Formed by Conducting-Probe Atomic Force Microscopy.* The Journal of Physical Chemistry B, 2005. **109**(35): p. 16801-16810.

12. Wang, X., et al., *High Seebeck coefficient from isolated oligo-phenyl arrays on single layered graphene <i>via</i> stepwise assembly.* Journal of Materials Chemistry C, 2023. **11**(42): p. 14652-14660.
13. Alaboson, J.M.P., et al., *Seeding Atomic Layer Deposition of High-k Dielectrics on Epitaxial Graphene with Organic Self-Assembled Monolayers.* ACS Nano, 2011. **5**(6): p. 5223-5232.
14. Liu, Y., et al., *Charge transport through molecular ensembles: Recent progress in molecular electronics.* Chemical Physics Reviews, 2021. **2**(2).
